# Study on the design calculation method and construction control method of suction drum foundation for guided frame platform

**DOI:** 10.1038/s41598-024-52068-6

**Published:** 2024-01-16

**Authors:** Zhang Xin, Li Yuansong, Liu Mingyue, Ye Shaoqi, Zhao Yong, Li Zhaoxian

**Affiliations:** 1 School of Civil Engineering and Architecture, Wuhan Institute of Technology, Wuhan, 430074 Hubei China; 2 The 5th Engineering Co., Ltd, China Railway Major Bridge Engineering, Jiujiang, 32000 Jiangxi China

**Keywords:** Civil engineering, Physical oceanography, Design, synthesis and processing

## Abstract

Currently, a standardized design and calculation specification for suction drum foundations has yet to exist in China. The engineering design currently depends mainly on the subjective understanding and engineering experience of the designers, which can be considered somewhat blind and subjective. In this paper, we utilize the offshore wind power project in Yangjiang City, Guangdong Province, as our case study. Building upon domestic and international research results, relevant investigations, design specifications, and engineering applications in related fields, we conduct a systematic study on the design calculation and construction control technology of the suction drum foundation. The document presents the design calculation and inspection of the suction drum foundation. Building on this foundation, we propose a sinking feasibility analysis method and a parameter value method for the suction drum foundation calculation. We also examine the suction drum foundation construction process, examining its control parameters, technology, and standards. Finally, based on the measured data from six four-barrel guided frame platform suction drum foundations that were successfully installed, the proposed design and control method are evaluated, and their effectiveness is verified. The results of this study can provide valuable references for the design and construction of similar suction drum foundation platforms.

## Introduction

As a new offshore engineering foundation structure, the suction drum is divided into single-drum and multi-drum foundations. A suction drum foundation is also known as a suction pile, suction cylinder, suction anchor, caisson foundation, barrel-shaped foundations, etc., which is named because of its appearance and shape of an inverted bucket and sinking through by suction. It has the advantages of a wide range of applications, short offshore construction time, reusable and low cost, and a vast application prospect^1^.

Sinking of suction drum foundations to the design depth and obtaining adequate bearing capacity are two critical issues in the design and use of drum foundations^2,3^. However, the prediction of the sinking resistance^4–6^ and the stability analysis of the soil inside the drum^7–9^ are the core elements of the suction drum foundation sinking to the design depth, and closely related to this is the prediction and calculation of the minimum required suction force and the maximum permissible suction force exerted in the suction drum^10,11^. The API^12,13^ and DNV^14,15^ pointed out that the bucket sinking resistance can be back-calculated by the minimum demanded suction force for negative pressure installation and gave the calculation method of the allowable suction force from the point of view of preventing reverse bearing capacity damage of the soil at the end. He et al.^16^ summarised the resistance calculation formulae in bucket foundation sinking at home and abroad. They proposed a weighted integrated method to predict the sinking resistance. Houlsby et al.^8,9^ proposed the calculation formulae for the suction bucket sinking resistance and the value of the negative pressure in clay and sandy soil. Andersen et al.^17,18^ proposed two calculation methods for the bucket penetration resistance in sandy soil, which are the bearing theory based on the friction angle of sandy soil and the empirical formula based on the strength of the CPT cone tip through the indoor model, field large scale test and the actual measurement data of the suction bucket in the stage of negative pressure penetration in the actual engineering. They systematically analyzed the value of the relevant empirical coefficients. Another concern in the process of sinking and penetration of the suction drum foundation is the destabilization damage of the soil inside the drum, in which the bulging soil plug prematurely contacts the inner wall of the drum lid and prevents it from being sunk and penetrated to the predetermined depth^19,20^. For the soil plug phenomenon in the negative pressure installation of the suction drum foundation, no more mature engineering measures have been seen so far that can effectively inhibit the instability of the soil plug and ensure that the drum is penetrated to the predetermined depth. The research on the bearing characteristics of suction buckets is mainly based on modifying the traditional foundation bearing theory. Eid^21^, through physical model test and numerical analysis, compares the vertical bearing capacity of sand foundation in the plate foundation, solid pier foundation, and barrel-shaped skirt foundation; the results show that the vertical bearing capacity of the barrel-shaped skirt foundation for the vertical bearing capacity of the solid pier foundation of 0.93 times, which is the reason for the bottom of the barrel skirt due to the stress concentration. Liu et al.^22^ used numerical analysis to study the bearing characteristics of barrel-shaped foundation in powder soil, obtained the foundation bearing capacity envelope of barrel-shaped foundation, and proposed a simplified calculation method of vertical bearing capacity and overturning stability of broad and shallow barrel-shaped foundation. Yan^23,24^ studied the vertical bearing characteristics of barrel-shaped foundations under vertical load by finite element calculation model and proposed the calculation method of discount factor applicable to the vertical bearing capacity of barrel-shaped foundations.

Monitoring and control of the construction process of suction drum foundations have been reported internationally^4,6,25^. However, due to the protection of intellectual property rights, the core technology is still rarely disclosed. Throughout the development history and technical status quo of suction drum foundations in the international arena^6,26^, the critical technologies for the construction control of suction drum foundations are summarised in three aspects: (1) calculation of penetration resistance and control index; (2) real-time monitoring and uploading of the control parameters; and (3) complete sets of installation equipment and control technologies. At present, through the research of some scholars, it is concluded that the monitoring and control of suction drum sinking penetration in wave and current environment is also a complicated problem that has not yet been well solved^5^. Some scholars made some achievements in telemetry remote control systems^27–29^. Three-barrel or four-barrel foundation is a common form of this kind of foundation, and how to ensure the synchronous sinking of each barrel in the process of sinking is still a critical technical problem to be solved urgently under the complex geological environment. In addition, settlement after completion of installation is a risk to any offshore structural foundation, and successful installation of foundations on site does not guarantee the subsequent overall stability of the structure under the severe wave conditions of global climate change and the sinking of offshore structural foundations is a significant risk faced by offshore infrastructures. International scholars such as Sumer^30^, Sassa^31^, and Miyamoto et al.^32^ do a series of cutting-edge research on the stability of permanent suction drum foundations.

The research on the sinking characteristics of suction drum foundations by scholars at home and abroad is mainly based on model tests, and the soil body is mainly single-layer homogeneous soil. There are relatively few studies based on field sinking data. Currently, the construction process and the calculation method of sinking resistance of suction drum foundations are mainly based on the design and construction standards formulated by European research institutes such as NGI and SPT. However, the geological conditions of China's Yellow Sea, East China Sea, and South China Sea are pretty different from those of the European North Sea, and the direct borrowing of design parameters and construction experience from European and American specifications may result in certain engineering risks or design redundancy. The fundamental reason why it is difficult to promote this new type of foundation structure is that (1) the actual application environment of suction bucket foundation (sea wind, waves, currents, etc.) is much more complex than the theoretical simulation environment, and the theoretical simulation can not be completely fitted with the actual environment in which the mechanical properties of the soil body change continuously with the depth of this characteristic; (2) the core technology in the process of sinking and penetration of the suction bucket foundation (the leveling control technology is not mature enough). Therefore, with the background of offshore wind power projects in Yangjiang City, Guangdong Province, this paper inverts and extrapolates the design calculation method of suction bucket foundation based on the measured data of six four-bucket guided rack platform suction bucket foundations successfully installed. The general formulae for penetration resistance of suction drum foundation in clay and cohesionless soil and the prediction formulae for self-weight penetration depth and suction penetration depth of suction drum foundation are obtained. At the same time, the new control technology of leveling (“Intelligent control system of suction penetration equipment integration system for accurate leveling” combined with “BIM platform for construction management”) is firstly applied to the process of sinking and penetration of suction drum foundation, so that the suction drum foundation of the guided frame platform can be smoothly penetrated to the design depth.

## Contents of suction drum foundation design and calculation methods

### Contents of suction drum foundation design

As the basis for the offshore construction platform, the core aim of designing the guided frame suction drum foundation is to ensure safe sinking to the designated depth, fulfill the bearing capacity criteria, and enable rapid recovery for maximum work efficiency. Its temporary nature means no need to factor in cyclic load, instantaneous load, deformation, or settlement as permanent foundations. The design focus is primarily on:

1. Suction drum foundation dimensions must take several factors into account, including suction drum foundation diameter (D), entry depth (h), wall thickness (t), pile tip shape, spacing, number of barrels (determined by the superstructure foundation type), geometric characteristics, location, mud surface constraints, material strength (typically Q235 or Q355 steel), installation methods and other relevant parameters.
2. Determining the penetration depth is crucial for ensuring the safe installation of the suction drum foundation. Accurate predictions of the foundation's depth of penetration under both its own weight and negative pressure are necessary to ensure it reaches its design depth, withstands the maximum calculated bearing capacity and uplift force, and maintains a certain degree of safety factor.
3. Penetration Feasibility Analysis: The successful penetration of the drum's foundation to the necessary depth is contingent on the relationship between the critical suction force, allowable suction force, and the requisite suction force in the soil layer.
4. Calculation of Bearing Capacity and Stability. The bearing capacity calculation involves evaluating the transfer of load resistance between the pile-soil system and superstructure, assessing the sinking process, and considering the in-situ working conditions of the suction drum foundation. Additionally, the foundation's strength and stability will be assessed.

### Calculation of sinking resistance of suction drum foundation

**Figure 1:**
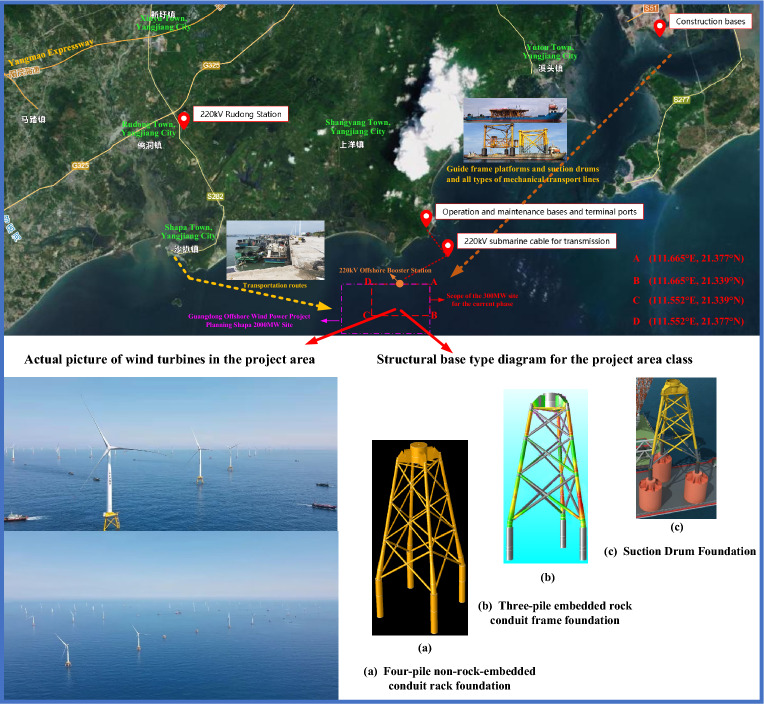
Offshore wind project suction drum foundation location map (drawings, photographs, and maps are derived from the project works by the author of this article.)

Penetration resistance calculation is an integral part of suction drum foundation design and a critical technical link in the construction control process. Presently, domestic and foreign engineering design is mainly based on three kinds of static balance method (API), based-CPTU method, and Houlsby analysis method. The scope of application of different theoretical calculation methods may differ, and the calculation results may also appear significantly different or even contrary. This paper combines the characteristics of the soil layer as well as the actual construction monitoring data to judge the applicability of the three methods in the offshore wind farm project in Xiangxi County, Yangjiang City, Guangdong Province. Figure 1 shows the specific site of the project area.

**Figure 2:**
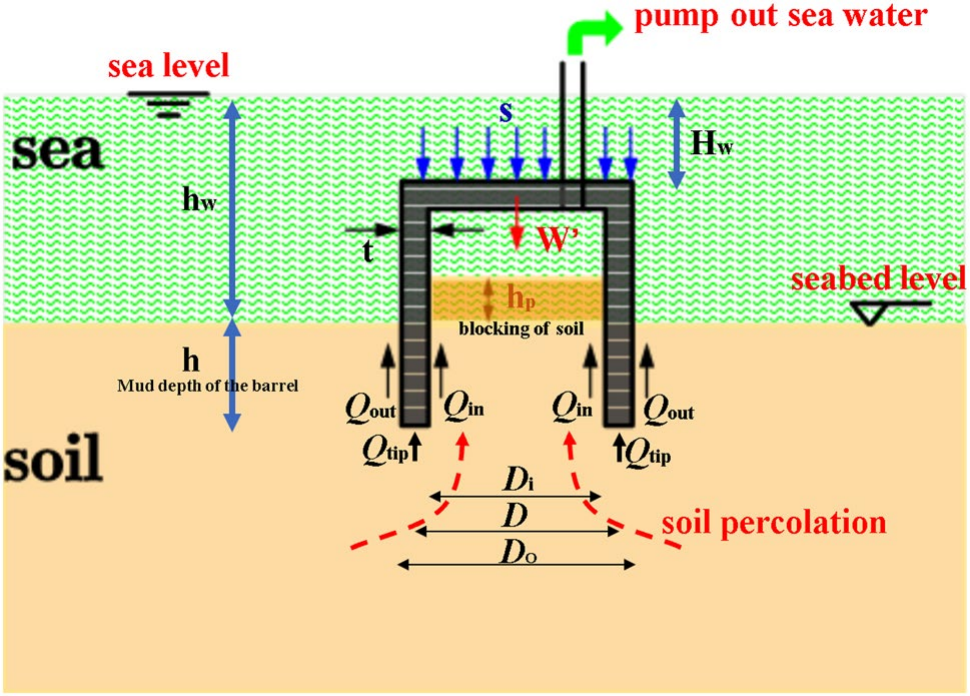
Force diagram of suction drum foundation sinking through.

Assuming that the foundation of the suction drum always remains vertical during the sinking process and there is no lateral shift or rotation, the analysis of the forces involved in the sinking process of the suction drum foundation is illustrated in Fig. 2. The main loads in the calculation are the underwater effective weight of the drum foundation, $W^{\prime }$; the friction force of the inner side of the suction drum foundation, *Q*_in_; the friction force of the outer side of the suction drum foundation, *Q*_out_; the friction force of the suction drum foundation at the end of the suction drum foundation, *Q*_tip_; and the driving force during sinking, F. The driving force is determined by the suction force value, *S*, multiplied by the inner wall of the suction drum foundation, *A*_in_. The sinking of the suction drum foundation is classified into self-weight penetration and negative pressure (suction) penetration, and the suction drum foundation can be sunk using Eqs. (1) and (2).

1 $$ {\text{Self-weight penetration}}:\;\quad W^{\prime } - Q_{{{\text{tot}}}} = W^{\prime } - Q_{{{\text{side}}}} - Q_{{{\text{tip}}}} = W^{\prime } - Q_{{{\text{in}}}} - Q_{{{\text{out}}}} - Q_{{{\text{tip}}}} \ge {0} $$

2 $$ {\text{suction penetration}}:W^{\prime } + F - Q_{{{\text{tot}}}} = W^{\prime } + F - Q_{{{\text{side}}}} - Q_{{{\text{tip}}}} = W^{\prime } + F - Q_{{{\text{in}}}} - Q_{{{\text{out}}}} - Q_{{{\text{tip}}}} \ge {0} $$

1. The static equilibrium method (API code method)^12,13^ is employed to determine the ultimate bearing capacity of the suction drum foundation. This method is based on the ultimate bearing theory, which views the penetration process as a slow and constant downward movement that surpasses the ultimate bearing capacity. At a certain depth, the ultimate bearing capacity of the foundation is equal to its penetration resistance. Based on the limit equilibrium theory of the rigid-plastic body model, assuming equal friction coefficients of the inner and outer suction drum sidewalls and disregarding the soil plug effect, Eq. (3) provides the penetration resistance *Q*_tot_ for penetration depth *h*_*n*_.
  3 $$ Q_{{\text{tot}}} = Q_{{\text{side}}} + Q_{{\text{tip}}} = \sum\limits_{i = 1}^{{\varvec{n}}} {\pi {\varvec{D}}_{0} \Delta h_{i} } \cdot ({\varvec{\alpha}}_{{i{\text{ns}}}} S_{{\text{u}i}} + K_{{\varvec{i}}} {\varvec{\gamma}}^{\prime}h_{i} \text{tan}{\varvec{\delta}}_{i}){ + }(N_{{\text{c}{\varvec{i}}}} S_{{\text{u}{\text{tip}}}}^{AVE} + N_{{\text{q}{\varvec{i}}}} {\varvec{\gamma}}{\prime} \, h_{{\varvec{n}}} ) \cdot A_{{\text{tip}}} $$

In Eq. (3), n represents the total number of calculated layers;* Q*_tot_ refers to the total penetration resistance (in kN); *Q*_side_ is the resistance along the barrel's side wall (in kN), and *Q*_tip_ is the end resistance of the barrel (in kN). *A*_tip_ denotes the area of the barrel end circle (in m^2^). *S*_ui_ represents the undrained shear strength of the soil in the ith sublayer (in kPa), while *N*_ci_ is the load-bearing capacity coefficient. The value of *N*_ci_ is related to the calculation's purpose and can be found in Table 1. *N*_qi_ represents the dimensionless bearing capacity coefficient. Table 2 for the corresponding value range. $S_{{{\text{utip}}}}^{AVE}$ represents the average undrained shear strength of the soil at the conclusion of the barrel (in kPa). *K* represents the lateral pressure coefficient (horizontal and vertical effective positive stress ratio). Usually, use measured values. If no measured values are available, the coefficient of earth pressure at rest, *K*, is assumed to be 0 for cohesive soils and 0.8 for non-cohesive soils or is calculated as *K* = (1-sin*φ*) where *φ* is the internal friction angle of the soil layer. $\gamma^{\prime }$ is the bulk density of the soil (kN/m^3^); *δ* is the angle of friction between the drum and the soil (°); *α*_ins_ is the cohesion coefficient of the soil, which is the inverse of the sensitivity of the soil (α_ins_ = 1/*S*_t_), *α*_ins_ takes the value of the range generally between 0.2 and 0.5, up to 1.0, and the specific value is calculated according to Eq. (4).

4 $$ a_{ins} = \left\{ \begin{gathered} \, 1 \, \psi \le {0}{\text{.25 }} \hfill \\ 0.5\psi^{ - 0.5} { 0}{\text{.25}} < \psi \le 1.0 \hfill \\ 0.5\psi^{ - 0.25} \, \psi > 1.0 \hfill \\ \end{gathered} \right. $$

**Table 1:** Recommended ***N***_**ci**_ coefficients.

Purpose of the solution	surface shape	***N***_**c**_	Notification Bulletin
Solving for pile end resistance	Square-shaped body	7.5	
Solving for the ultimate vacuum that causes soil plug failure	Orbicular	6.2 $\sim$ 9.0	Based on pile penetration rate
Solving for the sinking resistance of a protrusion	Variant	5 $\sim$ 13.5

**Table 2:** ***N***_**γ**_**, *N***_**q**_**, *N***_**c**_ coefficients.

*φ*(°)	*N*_c_	*N*_q_	*N*_γ(H)_	*N*_γ(v)_	*φ*(°)	*N*_c_	*N*_q_	*N*_γ(H)_	*N*_γ(v)_
0	5.14	1.00	0	0	24	19.33	9.61	6.90	9.44
2	5.69	1.20	0.01	0.15	26	22.25	11.83	9.53	12.54
4	6.17	1.43	0.05	0.34	28	25.80	14.71	13.13	16.72
6	6.82	1.72	0.14	0.57	30	30.15	18.40	18.09	22.40
8	7.52	2.06	0.27	0.86	32	35.50	23.18	24.95	30.22
10	8.53	2.47	0.47	1.22	34	42.18	29.45	34.54	41.06
12	9.29	2.97	0.76	1.69	36	50.61	37.77	48.08	56.31
14	10.37	3.58	1.16	2.29	38	61.36	48.92	67.43	78.03
16	11.62	4.33	1.72	3.06	40	75.36	64.23	95.51	109.11
18	13.09	5.25	2.49	4.07	42	93.69	85.36	136.72	155.55
20	14.83	6.40	3.54	5.39	44	118.41	115.35	198.77	224.64
22	16.89	7.82	4.96	7.13	45	133.86	134.86	240.95	271.76

Equation (4) specifies that the value of constraint *a*_ins_ cannot exceed 1. $\psi$ is defined as the ratio of *S*_*u*_ and $P_{O}^{\prime }$, where $P_{O}^{\prime }$ represents the effective vertical stress at the point of calculation.

This method does not consider the effect of negative pressure (suction), lateral shear stress, and other factors on the end-bearing capacity of suction drum foundations, which may have a negligible effect on thin-walled suction drum foundations. The suction drums used in this project are thin-walled so that this theoretical calculation can be used.

(2) Based CPTU method, Considering the difficulty of obtaining physical parameters by static equilibrium method based on static contact, API RP 2GEO and DNVGL-RP-C212 propose the based CPTU method^13,15^ to meet the actual engineering requirements. The method establishes the relationship between lateral frictional resistance, end resistance, and cone tip resistance *q*_*c*_. The sink-trough resistance is shown in Eq. (5).
  5 $$ Q_{{{\text{tot}}}} = \pi D_{i} \int\limits_{0}^{H} {k_{f} } \cdot q_{c} (z)dz + \pi D_{o} \int\limits_{0}^{H} {k_{f} } \cdot q_{c} (z)dz + \pi Dtk_{p} q_{c} (H) $$

In Eq. (5), *k*_*f*_(z) and *k*_*p*_(z) are the subjective coefficients of side friction resistance and end resistance, respectively, which are related to *q*_*c*_. *q*_*c*_(z) refers to the average penetration resistance ratio within the influence of the barrel foundation as represented by z, with the value being in MPa and its recommended value demonstrated in Table 3.

**Table 3:** Recommended clay and sandy soil coefficients* k*_*p*_ and *k*_*f*_*.*

Soil types	Common values		Maximum values	
	*k*_*p*_	*k*_*f*_	*k*_*p*_	*k*_*f*_
Clays	0.4	0.03	0.6	0.05
Sandy soil	0.3	0.001	0.6	0.003

Note: For the value of *kp*, select a lower value for compact sand and shallow water and a higher value for loose sand and deep water.

**Figure 3:**
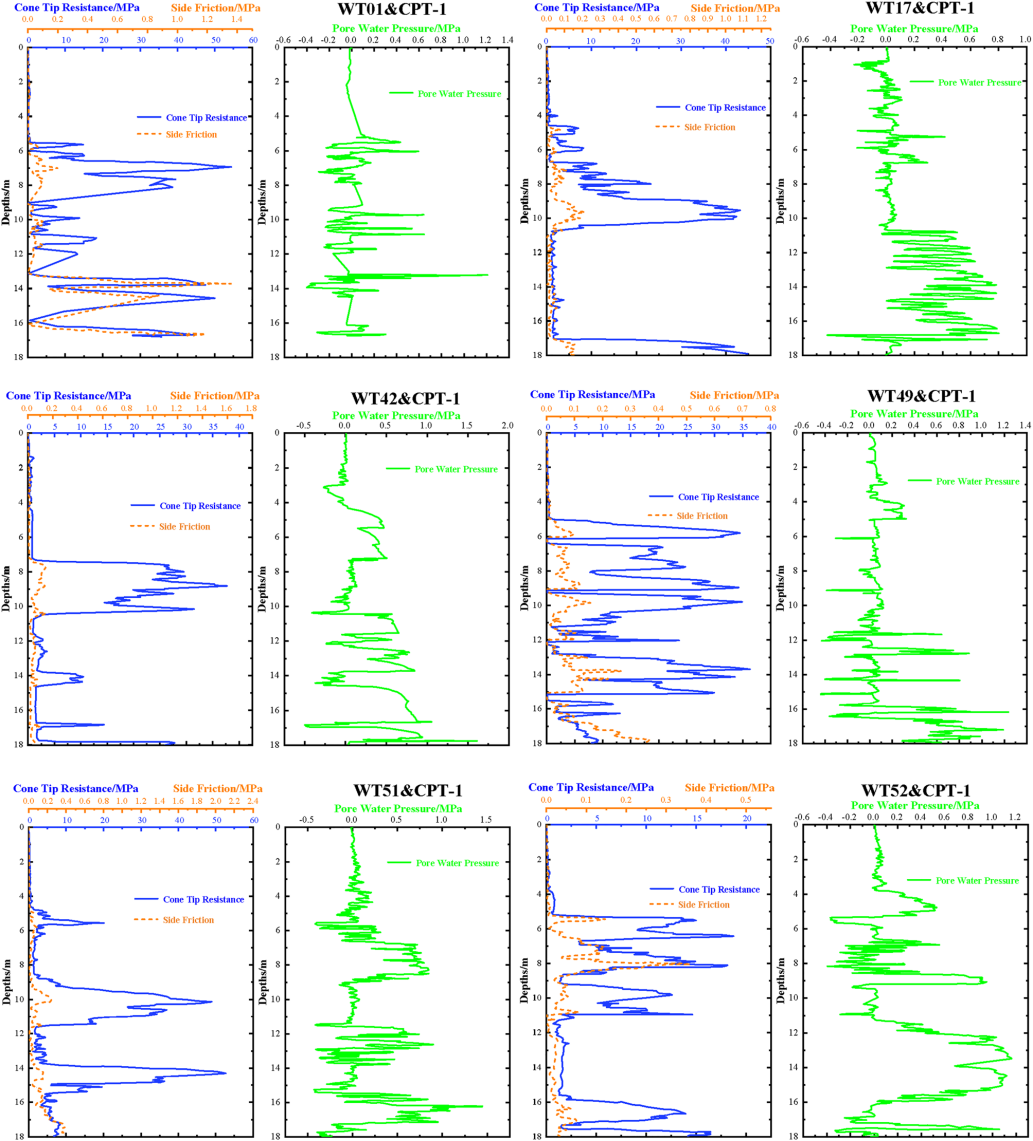
CPT survey data for WT01, WT17, WT42, WT49, WT51 and WT52.

The Based-CPTU method measures the static pressure penetration resistance. The suction drum foundation penetration process is unique and relies on negative pressure (suction) to drive the foundation sinking. Therefore, the empirical coefficient in Eq. (5) considers the interaction between seepage and the soil body by using the discount factor. This factor is typically obtained through large scale model tests or field tests. However, the empirical coefficient derived from the test exhibits a level of randomness and subjectivity. Furthermore, uncertainties arise in extrapolating the tip of the cone sinking resistance to other types of resistances, resulting in an ambiguous value for the empirical coefficient. It should be acknowledged that the method is precise and dependable when applied to the uniform soil examined in the test. Nonetheless, the range of *k*_*p*_ and *k*_*f*_ values is unpredictable in real-world engineering. Consequently, there are limitations to the practical application of this method, and the resultant sinking penetration resistance yields some deviation from actual outcomes. The distribution of cone tip resistance, lateral friction resistance, and pore water pressure along depth for the WT01, WT17, WT42, WT49, WT51, and WT52 machine locations in the project works is shown in Fig. 3 below.

(3) Houlsby's analytical method^8^ proposes a formula for calculating the resistance to sinking and penetration of suction barrel foundations in clays, assuming seepage is not considered and considering the different friction coefficients of the inner and outer walls of the barrel foundation. This formula is based on the foundation-bearing theory and the principle of moment equilibrium, as shown in Eq. (6).
  6 $$ Q_{{{\text{tot}}}} = ha_{o} s_{{{\text{u1}}}} {(}\pi D_{{\text{o}}} {) + }ha_{{\text{i}}} s_{{{\text{u1}}}} (\pi D_{{\text{i}}} ) + (\gamma ^{\prime}h - s + s_{{{\text{u2}}}} N_{c} )(\pi Dt) $$

In Eq. (6),* a*_o_ and *a*_i_ denote the adhesion coefficients of the external and internal walls of the drum, respectively. *D*_o_ and *D*_i_ represent the outer and inner diameters of the drum, while *s* refers to the negative pressure (suction). Additionally, *s*_u1_ is the average undrained shear strength of the soil, and *s*_u2_ indicates the undrained shear strength of the soil at the foundation's end.

The test results indicate that the Houlsby method's accuracy in predicting sinking resistance is dependent on the speed of suction drum foundation penetration. The lower the speed, the more accurate the sinking resistance value obtained. Conversely, higher speeds resulted in more significant deviations in the obtained values. The approach utilizes several parameters to anticipate sinking resistance precisely. For reliability, each parameter requires accuracy; hence, calculations with substantial deviations are unadvisable^33^.

### Depth prediction of suction drum foundation sinking penetration

Sinking the suction drum foundation is a challenging operation in marine engineering. The process involves two stages.

The initial phase is the self-weight penetration stage, during which the sinking platform is activated by self-weight exhaust. Open the four electric valves to initiate exhaust sinking and monitor the platform inclination closely. If the inclination deviates, shut down the corresponding side of the electric valve. Once the platform inclination falls within the control range, open all the electric valves for exhaust sinking. At this phase, it is crucial to ensure that a particular enclosed area can be created inside the barrel foundation once the weight of the foundation is applied onto the soft ground. Typically, a minimum depth of 0.5 m into the soft ground is necessary to meet this requirement, and the depth at which the weight settles into the ground can be calculated using Eq. (7).

7 $$ \begin{gathered} \sum\limits_{i = 1}^{n} {2\pi \Delta h_{{\text{i}}} D_{o} } a_{{{\text{ui}}}} s_{{{\text{ui}}}} + (N_{{{\text{ci}}}} \cdot s_{{{\text{utip}}}} + N_{{{\text{qi}}}} \sigma_{{\text{n}}}^{\prime } ) \cdot A_{{{\text{tip}}}} - W^{\prime } = 0 \hfill \\ L_{{{\text{SWP}}}} = \sum\limits_{i = 1}^{n} {\Delta h_{{\text{i}}} } \hfill \\ \end{gathered} $$

In Eq. (7), the layer height of layer i is represented by $\Delta$*h*_i_. We also use *s*_ui_ to denote the undrained shear strength of layer i, and *a*_ui_ to represent the cohesion coefficient of layer i. *D*_o_ indicates the outer diameter of the suction drum foundation, while *N*_ci_ and *N*_qi_ stand for the bearing capacity coefficients of layer i. Also, *L*_SWP_ represents the depth of penetration of the suction drum foundation under its weight.

Stage 2: Suction (negative pressure) penetration stage, during which the applied suction force creates pressure at the top of the drum foundation to provide a downward penetration force to help the foundation sink to the desired penetration depth. The suction penetration depth should be sufficient to ensure that the drum foundation has adequate bearing capacity, and the suction penetration depth can be predicted by Eq. (8).

8 $$ \begin{gathered} \sum\limits_{i = 1}^{n} {2\pi \Delta h_{{\text{i}}} D_{o} } a_{{{\text{ui}}}} s_{{{\text{ui}}}} + (N_{{{\text{ci}}}} \cdot s_{{{\text{utip}}}} + N_{{{\text{qi}}}} \sigma_{{\text{n}}}^{\prime} ) \cdot A_{{{\text{tip}}}} - W^{\prime} - \Delta {\text{U}}_{{{\text{allow}}}} \cdot A_{{{\text{in}}}} = 0 \hfill \\ L_{{{\text{TSP}}}} = \sum\limits_{i = 1}^{n} {\Delta h_{{\text{i}}} } \hfill \\ \end{gathered} $$

In Eq. (8), $\Delta$U_allow_ represents the admissible suction force. *A*_in_ denotes the area of vertical pressure, and *L*_TSP_ indicates the depth to which negative pressure penetrates the suction drum foundation.

### Feasibility analysis of suction drum foundation sinking

A precise comprehension and efficient management of the dynamic correlation between the effective weight (including superstructure load) $W^{\prime }$, negative pressure (suction force) s, vertical pressure area *A*_in_, and penetration resistance *Q*_tot_ of the suction drum foundation during sinking and penetration is fundamental for ensuring a seamless suction drum foundation sinking process. Equation (2) satisfies the correlation equation of each variable.

The force of suction $\Delta$U_req_ needed for the foundation of the suction drum to pierce through any depth is alternatively known as “the pressure required to pierce through to the stipulated depth.” Eq. (9) is used to determine the value of $\Delta$U_req_.

9 $$ \Delta {\text{U}}_{{{\text{req}}}} { = }\frac{{Q_{{{\text{tot}}}} - W^{\prime } }}{{A_{{{\text{in}}}} }} $$

The upper safe suction value, also known as the permissible suction force ($\Delta$U_allow_), is the maximum suction force that can be applied to the base of the suction drum. It is calculated using Eq. (10).

10 $$ \Delta {\text{U}}_{{{\text{allow}}}} { = }\frac{{\Delta {\text{U}}_{{{\text{crit}}}} }}{k} $$

The highest suction achievable for feasible construction can be determined using Eq. (11).

11 $$ \Delta {\text{U}}_{{{\text{allow}}}} {\text{ = min}}\left( {\frac{{\Delta {\text{U}}_{{{\text{crit}}}} }}{k},\;\gamma_{{\text{w}}} h} \right) $$

Equations (10) and (11): *k* is the safety factor, generally at least 1.25. It is recommended to take the value of 1.5. $\gamma_{{\text{w}}}$ is the seawater capacity (kN/m^3^). *h* is the distance from sea level to the top of the suction drum foundation (m), and $\frac{{\Delta {\text{U}}_{{{\text{crit}}}} }}{k}$ is the maximum suction force that the soil body in the suction drum can withstand.

**Figure 4:**
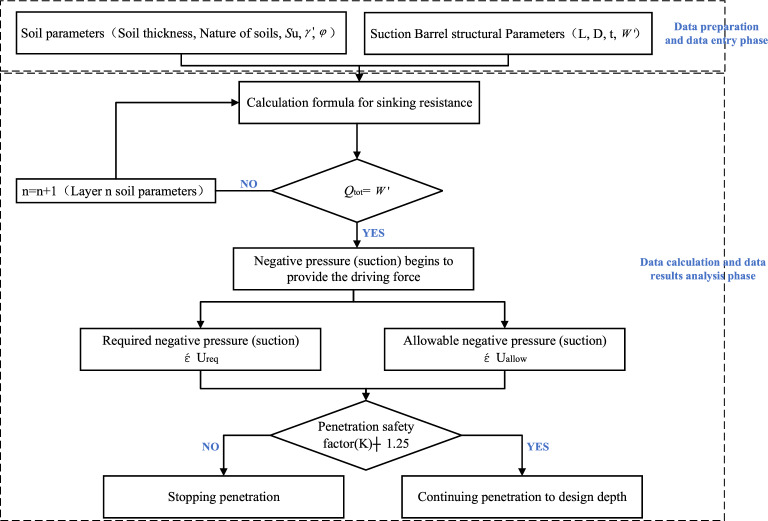
Feasibility process for suction drum sinking.

Penetration feasibility analysis is an important part that must be completed before construction, and it is also a way to anticipate safety risks in advance. Penetration feasibility analysis divides the soil layer into several thickness units within the designed penetration depth, calculates its critical suction, permissible suction, and demand suction, respectively, and judges whether the suction bucket can be safely penetrated layer by layer. According to engineering experience, each layer of soil is evaluated for penetrability, and treatment measures are taken for soil layers with a safety factor (K) of less than 1.25 to ensure that the suction drum foundation penetrates the design depth smoothly. According to the results of the investigation of soil quality for analysis, it should be noted that in the penetration feasibility analysis, the smaller the thickness of the soil layer divided into soil layers, the finer the calculation accuracy, but the corresponding workload is also more significant. According to the engineering experience, the general layering thickness is between 0.2 and 1.0 m. According to the experience of the suction drum foundation penetration analysis, it is considered that the layering thickness of 0.5 m can meet the requirements. The suction drum foundation penetration feasibility analysis process is shown in Fig. 4.

### Strength and stability calculation of suction drum foundation

Since most suction drum foundations comprise thin-walled steel drums, there is a potential for inadequate strength and local or overall instability during sinking penetration. For this reason, it is crucial to conduct calibration calculations to assess the strength and stability of the drum foundation structure. The suction drum foundation strength check is split into two parts, as per API RP 2A recommendations.

Step 1: Calculating stress comprises the calculation of axial stress *f*_*z*_, bending stress *f*_*x*_, and circumferential stress *f*_*y*_. Equations (12), (13), and (14) demonstrate the precise calculations.

12 $$ {\text{Under axial force}},{\text{ the axial stress}}\;f_{z} :f_{z} = \frac{P}{{\pi D_{o} t}} $$

13 $$ {\text{Under the bending moment force}},{\text{ the bending stress}}\;f_{x} :f_{x} = \frac{4M}{{\pi D_{o}^{2} t}} \cdot K_{x} $$

14 $$ {\text{Under }}\;{\text{the }}\;{\text{annular }}\;{\text{force,}}\;{\text{ the}}\;{\text{ annular}}\;{\text{ stress}}:f_{y} = \frac{{pD_{o} }}{2t} $$

Equations (12), (13), and (14), where *P* is the axial pressure, *D*_o_ is the outer diameter of the suction drum, *t* is the thickness of the suction drum wall, *M* is the bending moment of the central axis at the top of the drum foundation, *K*_*x*_ is the correction coefficient, typically taken as 1.0, and *p* is the circumferential force.

Step 2: Strength calibration, usually done according to Eq. (15).

15 $$ \begin{gathered} \, UC = \frac{{\sigma_{m} }}{{\sigma_{a} }},\sigma_{a} = \eta F_{y} \hfill \\ \sigma_{m} = \sqrt {(\sigma_{1} - \sigma_{2} )^{2} + (\sigma_{2} - \sigma_{3} )^{2} + (\sigma_{3} - \sigma_{1} )^{2} } = \sqrt {(f_{z} + f_{x} - f_{y} )^{2} + f_{y}^{2} + (f_{z} + f_{x} )^{2} } \hfill \\ \end{gathered} $$

In Eq. (15), the allowable stress ($\sigma_{a}$) should be compared to the Mises stress ($\sigma_{m}$), with the yield stress (*F*_*y*_) factored in and a discount factor of 0.8 ($\eta$) applied. A stress ratio *UC* value less than 1.0 is safe, and vice versa does not meet the requirements.

Due to the unique design of suction drum foundations, local plate and shell instability or general column instability may occur during sinking. This situation arises from the geometry of the drum foundation and the critical structural parameter, the thickness-to-diameter ratio (D/t). The current structural steel design standard (GB 50,017–2017) lacks a clause providing guidance on the stability calculations for drum shapes. As a result, the calculations can only be based on steel drums with 60 $\le$ D/t $<\hspace{0.17em}$300, t $\ge$ 5 mm, as stated in the API RP 2A-2005 code. However, for steel drums falling within the 300 $\le$ D/t $<$ 1200 range, t $\ge$ 6 mm, one must refer to the API BULL 2U code.

Local stability calculation is divided into five parts. Firstly, the stresses *f*_*z*_, *f*_*x*_, and *f*_*y*_ must be calculated under a single load. Secondly, the critical yield stress needs to be calculated under a single load. Thirdly, under a combination of loads, the critical stresses must comply with the provisions of Eq. (16) Fourthly, the allowable stress under buckling conditions should be calculated, with details provided by Eq. (17) and Parameter Table 4. Fifthly, the process of buckling stress calibration should be carried out, with details provided by Eq. (18).

16 $$ R_{a}^{2} - cR_{a} R_{h} + R_{h}^{2} = 1.0 $$

**Table 4:** Factor of safety for allowable stress design methods.

Design conditions	Role classification			
	Axial tension	bending force	Axial pressures	Cyclic pressures
Adoption of basic permissible stresses, e.g. taking into account the structural configuration, or effects that do exist during use	1.67	*F*_*y*_/*F*_*ax*_	1.67–2.0	2.0
Increase allowable stress by 1/3, e.g., consider wind loads, etc	1.25	*F*_*y*_/1.33*F*_*ax*_	1.25–1.5	1.5

Buckling stress safety factor: FS = 1.67 $\psi$ (normal condition); FS = 1.25 $\psi$ (extreme condition). Here, $\psi$ denotes the flexural stress safety factor. It is necessary to calculate the allowable stresses *F*_*az*_, *F*_*ax*_ and *F*_*ay*_.

17 $$ F_{az} = F_{ax} = \frac{{F_{\phi cj} }}{FS},\; \, F_{ay} = \frac{{F_{\theta cj} }}{FS} $$

18 $$ f_{z} + f_{x} \le F_{az} , \, f_{y} \le F_{ay} $$

In Eq. (16), *R*_*a*_ and *R*_*h*_ denote the ratio of stresses that arise from combined loading. The quantity *c* represents the influence coefficient of the barrel structure, while *φ* and $\theta$ refer to the axial and annular directions, respectively. For detailed explanations and calculation methods of the aforementioned physical quantities, refer to API Bulletin 2U^34^.

The stability calculation consists of two parts. Firstly, the column buckling stresses are calculated based on API RP 2A^12^. Secondly, Eqs. (19) and (20) are used to calculate the permissible stresses for column instability for each of the two cases.

19 $$ f_{{\text{z}}} /F_{{{\text{az}}}} 0.15,\;\frac{{f_{z} }}{{F_{az} }} + \frac{{f_{x} }}{{F_{ax} }} \le 1.0 $$

20 $$ f_{{\text{z}}} /F_{{{\text{az}}}} 0.15,\;\frac{{f_{z} }}{{F_{az} }} + \frac{{f_{x} }}{{F_{ax} }}\left( {\frac{{C_{m} }}{{1 - f_{z} /F_{e}^{\prime } }}} \right) \le 1.0 $$

Equation (20): *C*_*m*_ represents the bending moment coefficient equivalent, with a value of 0.85 when exposed to end restraints and 1.0 in reverse. $F_{e} ^{\prime}$ denotes the elastic instability allowable stress’s base value.

### Four-barrel guided frame platform installation test analysis

Using the six foundation positions WT01, WT17, WT42, WT49, WT51, and WT52 of the guide frame suction drum as an example, this study evaluates the suitability of the design values for the suction drum foundation of the guide frame platform by comparing them with measured data from the six positions.

Calculation parameters of suction drum foundation: The suction drum has an outer diameter of 5.0 m and a wall thickness of 0.03 m. The design penetration depth is 6.5 m, and the self-weight of the drum body is 498.48 kN, with an additional underwater self-weight of 410.55 kN. The steel is Q235, modulus of elasticity *E* = 210 GPa, Poisson's ratio *μ* = 0.3, yield strength *σ*_y_ = 225 MPa, vertical force at pile top during penetration is 766 kN, vertical load at in-situ condition is 1743 kN, bending moment M = 669 kN·m, and maximum horizontal load is 378 kN.

The foundation stratigraphy's calculated parameters for the suction drums of the WT01, WT17, WT42, WT49, WT51, and WT52 guided frame platforms are presented in Tables 5, 6, 7, 8, 9 and 10.

**Table 5:** Stratigraphy and calculation parameters for the pile location of the WT01 platform.

$${\text{Soil Classification}}$$	$${\text{Depth of Stratum}}$$ (m)	$${\text{Effective Heavy}}$$$$\gamma ^{\prime}$$/(kN/m^3^)	$${\text{Undrained Shear Strength}}$$ *S*_u_/(kPa)	$${\text{Internal Friction Angle}}$$ *φ*/(°)
①_1_ Silty clay	3.3	7.4	6.0	
①_2_ Silty clay	4.1	6.9	25.0	
①_2_ Powdery clay	5.0	6.9	25.0	
②_4_ Powdery clay	11.6	7.4	42.0	
②_5_ Clay	16.2	8.4	78.0

**Table 6:** Stratigraphy and calculation parameters for the pile location of the WT17 platform.

${\text{Soil Classification}}$	${\text{Depth of Stratum}}$ (m)	${\text{Effective Heavy}}$ $\gamma ^{\prime}$/(kN/m^3^)	${\text{Undrained Shear Strength}}$ *S*_u_/(kPa)	${\text{Internal Friction Angle}}$ *φ*/(°)
①_1_ Mud	1.0	6.5	4.0	
①_2_ Powdery clay	4.6	8.5	20.0	
①_3_ Grit	6.2	10.9		25.0
①_3_ Clay	6.7	7.9	32.0	
②_1_ Fine sand	8.5	9.2		30.0
②_3_ Grit	10.6	9.5		38.0
②_4_ Powdery clay	14.0	8.0	55.0	
②_4_ Powdery clay	17.2	8.1	65.0

**Table 7:** Stratigraphy and calculation parameters for the pile location of the WT42 platform.

${\text{Soil Classification}}$	${\text{Depth of Stratum}}$ (m)	${\text{Effective Heavy}}$ $\gamma ^{\prime}$/(kN/m^3^)	${\text{Undrained Shear Strength}}$ *S*_u_/(kPa)	${\text{Internal Friction Angle}}$ *φ*/(°)
①_1_ Silty clay	2.1	7.2	8.0	
①_2_ Clay	4.2	7.6	30.0	
①_3_ Clay	7.0	7.2	49.0	
②_1_ Coarse sand	10.1	9.3		35.0
②_2_ Clay	11.4	8.6	62.0	
②_4_ Clay	13.8	9.6	100.0	
②_5_ Coarse sand	14.4	11.7		30.0
②_6_ Clay	17.6	7.7	88.0

**Table 8:** Stratigraphy and calculation parameters for the pile location of the WT49 platform.

${\text{Soil Classification}}$	${\text{Depth of Stratum}}$ (m)	${\text{Effective Heavy}}$ $\gamma ^{\prime}$/(kN/m^3^)	${\text{Undrained Shear Strength}}$ *S*_u_/(kPa)	${\text{Internal Friction Angle}}$ *φ*/(°)
①_1_ Silty clay	2.2	6.1	5.0	
①_2_ Clay	3.9	5.5	16.0	
①_2_ Clay	5.0	7.1	26.0	
②_3_ Grit	10.5	9.4		35.0
②_3_ Coarse sand	12.0	8.7		31.0
②_4_ Clay	12.8	7.5	50.0	
②_5_ Coarse sand	15.5	8.4		35.0

**Table 9:** Stratigraphy and calculation parameters for the pile location of the WT51 platform.

${\text{Soil Classification}}$	${\text{Depth of Stratum}}$ (m)	${\text{Effective Heavy}}$ $\gamma ^{\prime}$/(kN/m^3^)	${\text{Undrained Shear Strength}}$ *S*_u_/(kPa)	${\text{Internal Friction Angle}}$ *φ*/(°)
①_1_ Silty clay	2.2	7.0	7.0	
①_2_ Powdery clay	4.7	7.8	25.0	
②_1_ Grit	5.7	10.1		25.0
②_2_ Clay	6.4	8.7	115.0	
②_2_ Clay	8.6	8.7	80.0	
②_5_ Grit	11.6	10.0		35.0
②_4_ Powdery clay	13.1	10.5	90.0	
②_4_ Powdery clay	13.7	10.5	110.0	
②_6_ Grit	15.4	10.3		35.0

**Table 10:** Stratigraphy and Calculation Parameters for the Pile Location of the WT52 Platform.

$${\text{Soil Classification}}$$	$${\text{Depth of Stratum}}$$ (m)	$${\text{Effective Heavy}}$$$$\gamma ^{\prime}$$/(kN/m^3^)	$${\text{Undrained Shear Strength}}$$ *S*_u_/(kPa)	$${\text{Internal Friction Angle}}$$ *φ*/(°)
①_1_ Silty clay	1.0	7.9	4.0	
①_2_ Silty clay	4.0	8.1	10.0	
②_2_ Clay	5.3	8.2	41.0	
②_3_ Medium sand	8.6	8.7		27.0
②_3_ Clay	9.2	8.5	80.0	
②_3_ Coarse sand	11.0	8.8		25.0
②_4_ Clay	15.6	8.1	80.0	

**Figure 5:**
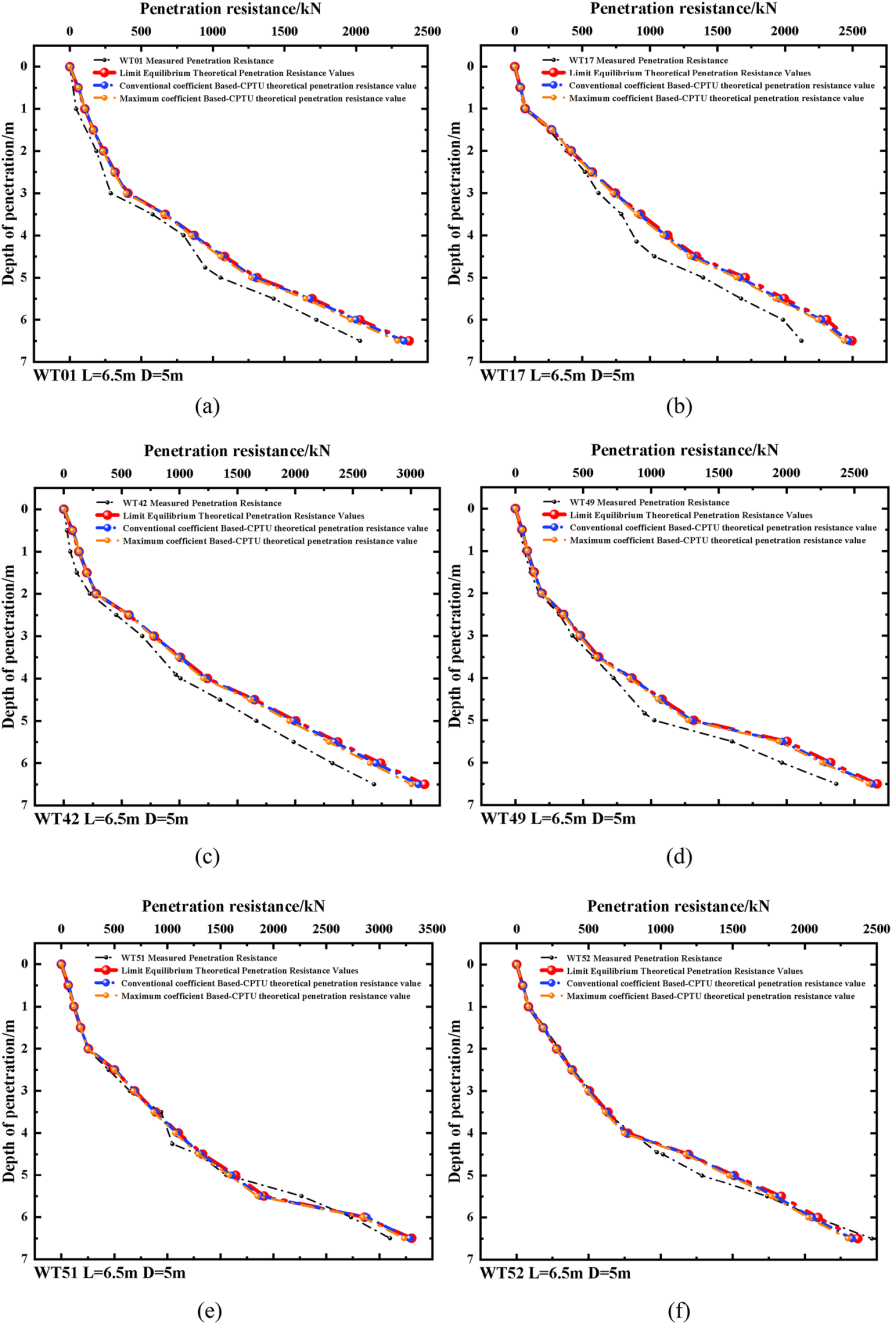
Resistance to penetration and depth of penetration curves.

Calculation of the penetration resistance was conducted based on the measured data from six machine positions. The resistance was calculated using both the static equilibrium and the Based-CPTU methods. The results of the data obtained from the four-barrel guided frame platform can be found in Fig. 5a through f.

From Fig. 5, it is evident that the difference between the theoretical and measured values is minimal. Even though the theoretical calculations do not consider the impact of seepage, the values obtained from the theoretical formulae demonstrate a high level of agreement with the measured data. Therefore, the theoretical forecast of the resistance to settling through the suction drum's foundation is valid and viable.

While theoretical calculations may be prone to error, the theoretical model provides a valuable tool for estimating suction drum foundation immersion resistance during actual construction. Such estimates are essential for effective planning and construction control. However, it is imperative to emphasize that actual construction also necessitates monitoring and adjustments to maintain the accuracy and safety of the sinking penetration process.

1. Prediction of penetration depth: The calculated predicted penetration depth was compared and analyzed with the measured penetration depth of six machine positions to predict penetration depth. Table 11 shows the results of the analysis.

**Table 11:** Comparative analysis of theoretical and measured values of penetration depth of suction drum foundation.

Suction Barrel Foundation	Theoretical *L*_SWP_ /(m)	Measured *L*_SWP_ /(m)	Error Value /(m)
WT01	4.71	4.76	+ 0.05
WT17	4.11	4.15	+ 0.04
WT42	3.86	3.91	+ 0.05
WT49	4.70	4.84	+ 0.14
WT51	4.16	4.26	+ 0.10
WT52	4.48	4.45	− 0.03

From Table 11, it is evident that predicting the penetration depth of the suction drum foundation is feasible with an error ranging between − 0.03 and + 0.14 m. This implies that the difference between the predicted theoretical value and the actual measured value is minor. Therefore, it can be inferred that the predictions of the suction drum foundation penetration depth are reasonably dependable and accurate. Such findings provide crucial information for engineers and construction personnel to reference and ground their operations on the predicted values during construction. Nonetheless, it is still advisable to closely monitor and adjust the penetration depth of the suction drum foundations during construction to confirm that they meet the design prerequisites. Real-time monitoring data can be utilized to make alterations and optimizations if needed.

**Figure 6:**
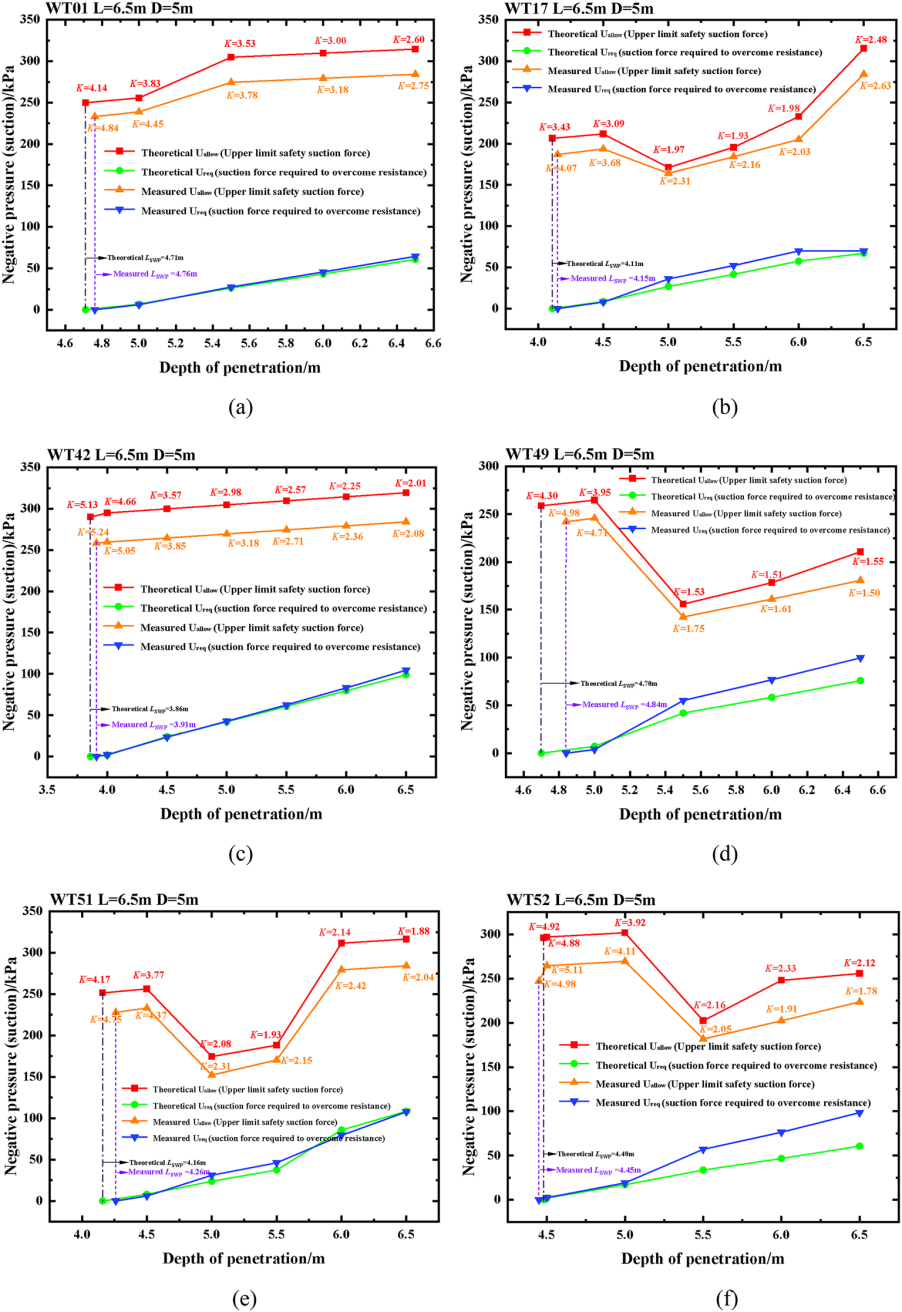
WT01, WT17, WT42, WT49, WT51 and WT52 machine position penetration feasibility analysis diagrams.

Penetration feasibility analysis has been conducted for the construction project of six suction drum sinking positions, specifically WT01, WT17, WT42, WT49, WT51, and WT52. The analysis calculations indicate that the foundation of the suction drum can be safely penetrated with a safety factor K estimation of K greater than or equal to 1.25. After analyzing and calculating, the results of the feasibility analysis for sinking suction drum foundations at the six positions above are displayed in Fig. 6a through f.

By examining Fig. 6a–f, it is evident that the actual necessary suction force exceeds the theoretically required suction force. This is necessary to ensure that the foundation of the suction drum can offer an adequate driving force for smooth sinking during negative pressure. However, the maximal negative pressure allowed for safety is higher than the negative pressure measured, which provides additional security to the construction and establishes a degree of redundancy between them to safeguard against damages or unforeseen circumstances. In the figure above, it is evident that the measured K value exceeds the theoretical K value, indicating a high level of safety in construction. Additionally, both K values are equal to or greater than 1.25. If a K value of less than 1.25 arises during construction, cyclic pressurization can drive the suction drum foundation to the intended depth.

(2) Strength calculation of suction drum foundation. The axial stress *f*_*z*_ determines the forces acting on the suction drum foundation, bending stress *f*_*x*_, and circumferential stress *f*_*y*_.The principal stresses are $\sigma$_1_ = *f*_*z*_ + *f*_*x*_, $\sigma$_2_ = 0 and $\sigma$_3_ = *f*_*y*_. Based on the fourth strength theory, the Mises equivalent stress is calculated. If the Mises stress is lower than the yield strength of the suction drum foundation, then no strength damage will occur to the suction drum foundation. The strength check results for the suction drum foundation are presented in Table 12. The stress ratio (*UC*) of Mises stress($\sigma$_m_) and allowable stress ($\sigma$_a_) in the suction drum foundations of WT01, WT17, WT42, WT49, WT51, and WT52 are all less than 1. This implies that the stress level of the suction drum foundation meets the specified strength standard and has good safety.
(3) Calculation of Suction Drum Foundation Stability: Firstly, analyze and calculate the axial stress *f*_*z*_ and circumferential stress *f*_*y*_ caused by local plate and shell buckling and overall column buckling of the foundation of the suction drum. Then, based on the combination of axial and circumferential loads, calculate the critical yield stress of the structure in both axial and circumferential directions and derive the safety coefficient. Take the minimum of the two coefficients as the allowable axial and circumferential stress of the structure. At the same time, determine the actual stress of the structure in the axial and circumferential directions caused by external forces and calculate the results. The calculated results must comply with the requirement that the allowable stress is greater than the actual stress. Table 13 displays the calculated results for the local stability and overall stability of the suction drum foundation.

**Table 12:** Strength calculation of suction drum foundation during sinking and penetration.

Pile Position	*f*_*z*_ /(MPa)	*f*_*x*_ /(MPa)	*f*_*y*_ /(MPa)	$\sigma$_m_/(MPa)	$\sigma$_a_/(MPa)	*UC*
WT01	6.857	2.767	12.668	16.198	180.000	0.09
WT17	7.183	2.767	13.972	17.618	180.000	0.10
WT42	8.840	2.767	20.602	25.299	180.000	0.14
WT49	7.643	2.767	15.813	19.688	180.000	0.11
WT51	9.336	2.767	22.585	27.684	180.000	0.15
WT52	6.852	2.767	12.648	16.177	180.000	0.09

**Table 13:** Stability calculations for suction drum foundations.

Pile Position	Localised plate and shell buckling						Integral columnar flexion			
	Axial stress /(MPa)	Allowable stress /(MPa)	UR	*f*_*y*_/(MPa)	Allowable stress /(MPa)	UR	*f*_*a*_/(MPa)	*f*_*b*_/(MPa)	Allowable stress /(MPa)	UR
WT01	9.516	67.971	**0.14**	12.668	63.340	**0.20**	6.857	2.767	68.743	**0.14**
WT17	9.668	96.685	**0.10**	13.972	82.188	**0.17**	7.183	2.767	66.333	**0.15**
WT42	11.479	57.395	**0.20**	20.602	68.673	**0.30**	8.840	2.767	61.089	**0.19**
WT49	10.189	66.162	**0.15**	15.813	68.752	**0.23**	7.643	2.767	67.161	**0.16**
WT51	12.160	51.966	**0.23**	22.585	68.439	**0.33**	9.336	2.767	56.556	**0.21**
WT52	9.343	99.394	**0.09**	12.648	84.320	**0.15**	6.852	2.767	71.252	**0.14**

Significant values are in [bold].

The stress ratio *UR* is less than 1, fulfilling the design requirements. To summarize, the suction drum foundation's overall structural design is sound and can satisfy the design requirements, ensuring a secure utilization of the suction drum foundation.

## Suction drum foundation construction control method

### Suction drum foundation sinking control parameters and control criteria

Suction drum foundation sinking control is a crucial technology in foundation construction. To maintain reasonable sinking speed and correct attitude during the suction drum foundation sinking process, it is vital to pay close attention to the characteristics of the soil layer, suction drum pressure difference both inside and outside, sinking speed, soil plugs, inclination, and other indicators. Adjust the sinking control method in a timely according to changes in indicators.

The parameters governing the sinking of the suction drum foundation comprise the following:

1. Suction limit values, encompassing critical suction (∆U_crit_), permissible suction (∆U_allow_), also referred to as the "upper safe suction value," and required suction (∆U_req_), denoting the "pressure required to penetrate to a specified depth." For reference, we have tabulated the relevant formulae in Table 14.
2. The successful installation of the suction drum foundation to the specified design depth relies on controlling the height of the soil plug. Equation (21) calculates the height of the soil plug.
  21 $$ H_{p}  = m\left( {\frac{{D_{o}^{2} }}{{D_{i}^{2} }} - 1} \right)h $$

**Table 14:** Limit value of suction force for suction drum foundation sinking through suction force.

Control parameters	Calculation formula
Critical suction (clay)	$\Delta {\text{U}}_{crit} { = }N_{c} \cdot S_{utip}^{AVE} + \frac{{A_{inside} \cdot (\alpha_{ins} \cdot S_{uDSS} )_{AVE} }}{{A_{in} }}$
Critical suction (non-cohesive soil)	$\Delta {\text{U}}_{crit} = \left\{ {\pi - \arctan \left[ {5(L/D)^{0.85} } \right]\frac{{2\left( {\pi - 1} \right)}}{\pi }} \right\}L\gamma^{\prime }$
Allowable suction power	$\Delta {\text{U}}_{{{\text{allow}}}} { = }\frac{{\Delta {\text{U}}_{{{\text{crit}}}} }}{k}$, $\Delta {\text{U}}_{{{\text{allow}}}} {\text{ = min}}\left( {\frac{{\Delta {\text{U}}_{{{\text{crit}}}} }}{k},\;\gamma_{{\text{w}}} h} \right)$
Required suction power	$\Delta {\text{U}}_{{{\text{req}}}} { = }\frac{{Q_{{{\text{tot}}}} - W^{\prime } }}{{A_{{{\text{in}}}} }}$

The straight shear strength (kPa) of clayey soil is represented by *SuDSS*, and the meanings of the symbols used in Eqs. (3), (9), (10), and (11) are explained within these equations.

In Eq. (21), *H*_*p*_ represents the height of soil plug augmentation, *m* denotes the parameter of soil plug augmentation, and *h* represents the depth of barrel subsidence. This formula was originally derived from a subsidence test of a suction drum foundation on sandy soil and subsequently validated through model testing, prototype testing, and finite element analysis to be applicable for calculating the height of soil plug augmentation for suction drum foundation subsidence on clay soil.

(3) The sinking rate must be controlled during the penetration of the suction drum guide frame foundation. If the sinking rate is too fast, it may generate additional pressure under the soil, while too low a rate will create negative pore water pressure due to soil extrusion, and both can lead to soil disturbance. The resistance to penetration is related to the penetration rate, whereby a higher rate of penetration results in more excellent resistance. The sinking rate and magnitude of the negative pressure are directly proportional; the greater the negative pressure, the faster the sinking rate.

The sinking rate is closely linked to pump flow, and controlling the sinking rate is mainly achieved through pump flow control. There are two main methods for controlling the pump flow: one is to adjust the frequency of the pump motor to alter the pump's speed and flow, known as indirect control; the second is to directly control the opening of the pump outlet pipe gate valve to control the pump flow, known as direct control. The former method is easily automated, while the latter yields better control results.

Due to the temporary nature of the suction drum foundation of the guide frame platform, construction control is determined by the following criteria:

1. The soil plug increase amount should not exceed 200 mm;
2. The actual suction force during the suction drum penetration must never exceed the flexion control line;
3. In the course of penetration, real-time monitoring of suction drum internal and external pressure differences, inclination, drum integrity, and other aspects should take place, and construction records should be produced;
4. Multi-barrel guided frame foundation penetration and sinking should be controlled by elevation; the foundation shall be penetrated to the design elevation;
5. Multi-barrel guided frame fan foundation penetration sinking should control the sinking rate: self-weight sinking stage: the foundation began to sink to the bottom of the foundation steel barrel from the mud surface 0.5 m, the sinking rate is not greater than 10 cm/min (6 m/h); the bottom of the foundation steel barrel from the mud surface 0.5 m to the foundation of the steel barrel into the mud 2 m, the sinking rate is not greater than 3 cm/min (1.8 m/h); the foundation of the steel barrel into the mud 2 m The sinking rate is not more than 5 cm/min (3 m/h) until the completion of the self-weight sinking stage. Negative pressure sinking stage: negative pressure sinking stage foundation sinking rate is not more than 2.5 cm/min (1.5 m/h);
6. Base penetration allowable deviation can be found in Table 15.

**Table 15:** Suction drum foundation penetration accuracy requirements.

Requests	Measurement parameters	Sinking accuracy values
1	Absolute position	< 500 mm
2	Elevation	− 500 $\sim$ 100 mm
3	Horizontal degree	$\le$ 2‰

### Suction drum foundation penetration control method

During the construction process, it is crucial to monitor the sinking process of the suction drum foundation in real time to ensure the safe and timely installation of the foundation. The intelligent control system has been adopted to automatically monitor the platform sinking process in the multi-drum guide frame. The control system monitors the platform level, sinking speed, and the negative pressure of the foundation of each suction drum during sinking. This ensures the safe sinking of the multi-drum guide frame platform to the expected depth. Technical abbreviations are explained on first use. The intelligent control system can automatically monitor and regulate the sinking process of the suction drum foundation, enhancing construction efficiency and safety. Real-time data acquisition and analysis facilitate automatic adjustment as per the preset control algorithm. This enables prompt identification and resolution of potential issues for ensuring the safe installation of the suction drum foundation and timely completion of the construction task.

In summary, implementing an intelligent control system to monitor the suction drum foundation sinking process in real time is an effective way to ensure construction safety and control progress. The use of this automated monitoring system can increase construction efficiency, diminish construction risks, and guarantee that the suction drum foundation is installed as per the design specifications.

**Figure 7:**
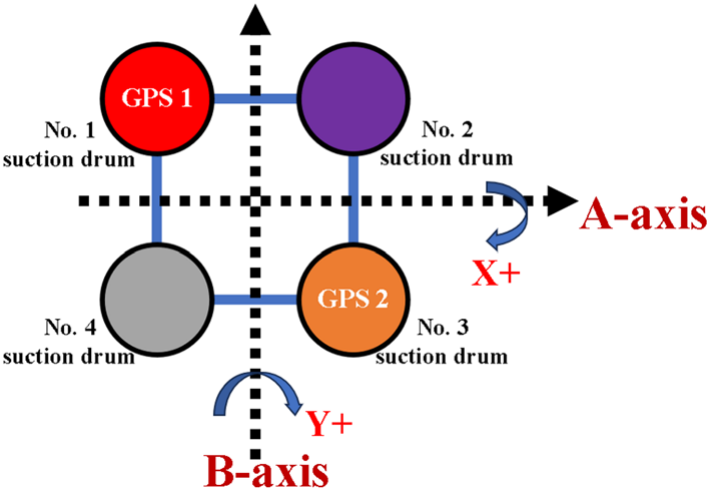
Definition of tilt angle direction.

1. Control Principle. The control system enables real-time monitoring of the platform tilt angle during the sinking process. As shown in Fig. 7, for illustration, the direction of the tilt angle is defined. The four-barrel guiding frame platform records the clockwise rotation around the A-axis as X+tilt angle and around the B-axis as Y+tilt angle. When the positive X tilt angle arises, barrels 1 and 2 ascend while barrels 3 and 4 descend. When the Y tilt angle becomes positive, barrels 1 and 4 rise while barrels 2 and 3 drop.

**Figure 8:**
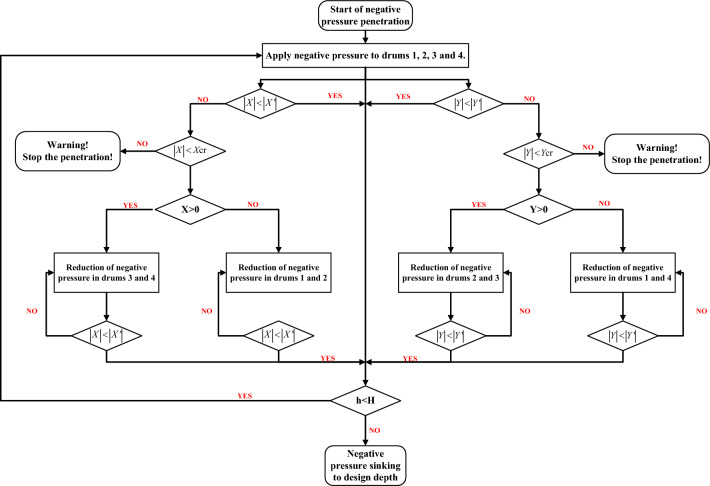
Control principle of automatic control system for four-barrel guided frame platform.

The control principle for the automatic control system of negative pressure sinking and penetration for the four-barrel guided frame platform is demonstrated in Fig. 8.

The figure above depicts the sinking principle through the suction drum foundation under negative pressure conditions. To begin, release the bungee block to initiate pumping and ensure the pressure difference in the four drums is nearly equivalent. After a period of penetration, the angles of inclination in the X and Y directions should be verified. It is required that both angles be within the DNV^35^ specifications for inclination angles $X^{\prime }$ and $Y^{\prime }$, as well as the limit values for inclination angles *X*cr and *Y*cr, or vice versa. The negative pressure of the suction drum foundation should be adjusted to achieve equilibrium. Next, monitor if the suction drum foundation reaches the design depth H. It is important to mention that the tilt angle limit is the maximum overall platform tilt angle permitted whilst leveling, which is *X*cr = *Y*cr =  ± 1° and DNV^35^ specification allows tilt angle $X^{\prime } = Y^{\prime } = \pm 0.25^\circ$. When sinking the platform of the multi-barrel guide frame, it is essential to refrain from increasing the negative pressure by pumping the high-level drums to prevent damage from seepage. Tilt adjustments can occur with the injection of water into the low-water-level drums, effectively decreasing negative pressure on the suction drums. Water injection serves to level the suction drums, and the passive lifting force results from the transfer of the filling suction drums' lifting force to the unfilled suction drums. In this process, a rigid structure is created by connecting each barrel of the platform through a steel pipe. The water-filled suction barrel then transfers the lifting force to the unfilled suction barrel via this pipe, generating a passive lifting force. It is crucial to balance the water levels and the transfer of lifting force between the drums when injecting water to maintain the platform’s stability, as stated in reference^36^.

**Figure 9:**
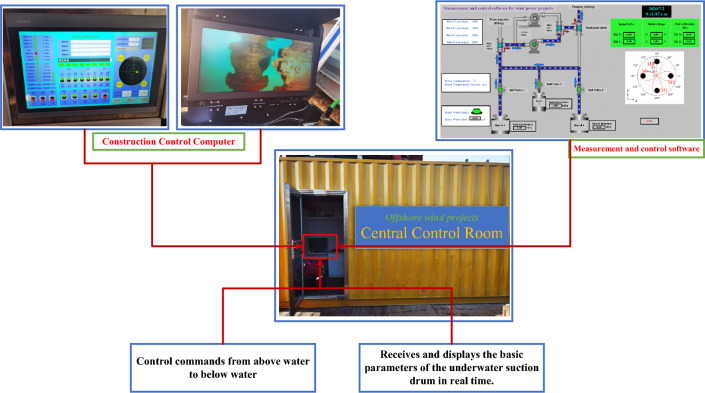
Control cabinet working principle.

(2) Monitoring Equipment. The intelligent suction drum negative pressure sinking control system comprises water pipelines, a pump skid, cable lines, and a control cabinet, amongst other equipment. The pump skid is mounted on a pre-set platform base while the control cabinet is on the floating crane. Figure 9 shows the working principle of the control cabinet, which is connected to the pump sled block via the cable. The intelligent control system utilizes an industrial computer and measurement and control software to transmit control commands to the underwater device through the control cabinet while concurrently receiving and displaying the measured parameters in real time. The water pipeline in each suction drum is linked to the intelligent control system. Water is pumped via the intelligent control system to the suction drum foundation to finalize the sinking and coherent installation of the multi-barrel guided frame platform.

The pump skid comprises four components: the electronic cabin, hydraulic mechanism, electric water pump, and locking mechanism. The electronic cabin is responsible for executing control instructions, monitoring, and transmitting state information of the suction drum foundation. The hydraulic mechanism provides mechanical power, including the hydraulic control valve and the pump interface locking mechanism. The locking mechanism is used to realize the locking and unlocking of the pump skid block and the suction drum foundation. The pumping volume of the electric water pump is remotely controlled to complete the negative pressure sinking of the suction drum foundation.

Such an intelligent control system can effectively regulate the inclination angle and speed of the suction drum foundation during the sinking process. Additionally, it can enhance the safety and efficiency of the construction process by monitoring and transmitting various parameters in real-time.

Using the “Construction Management BIM Platform” in conjunction with manual re-measurement and pump sled block measurement sensing system can mutually verify the positioning accuracy of the sinker installation, ensuring that the overall construction accuracy of the guiding frame platform aligns with the design requirements.

**Figure 10:**
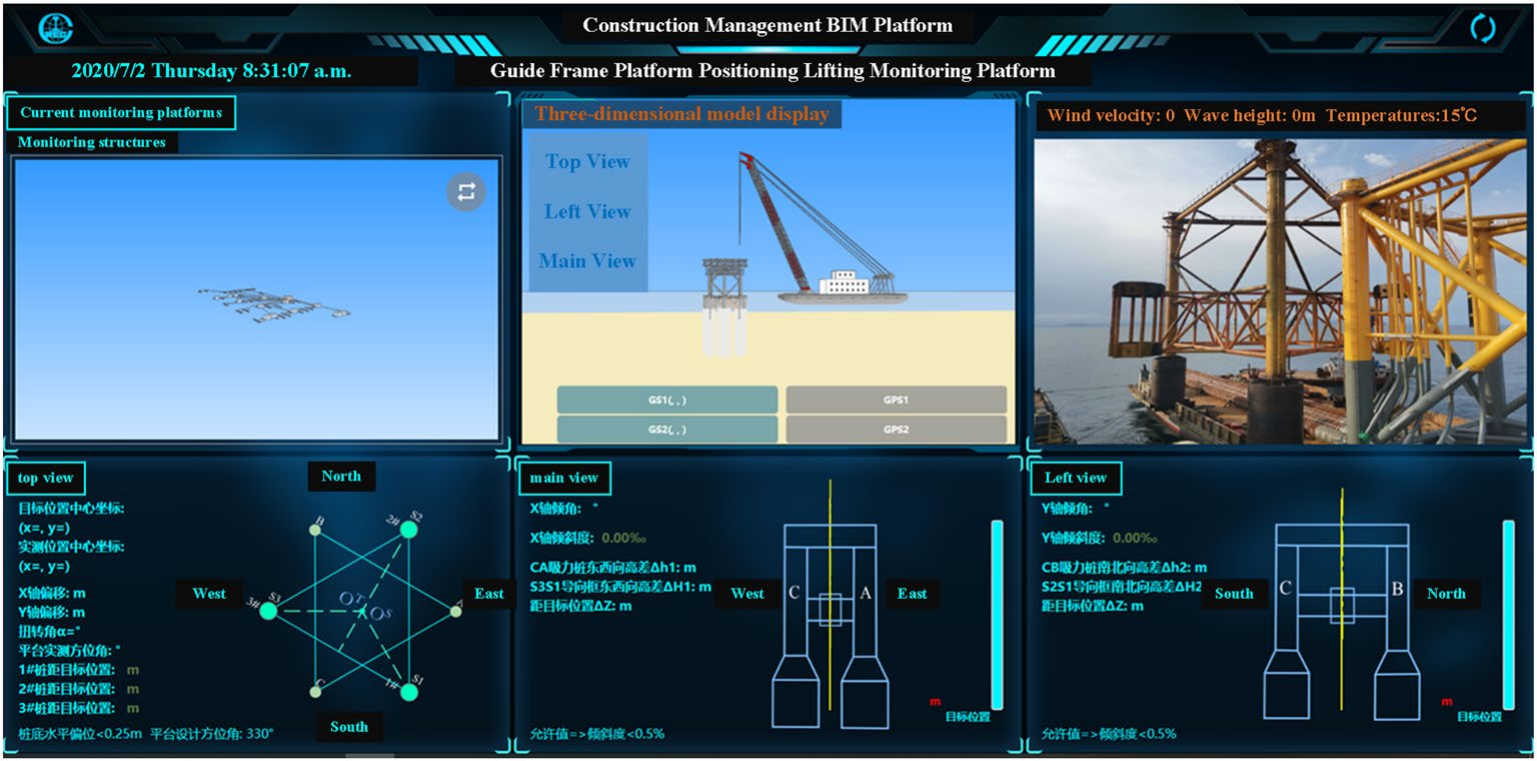
Construction management BIM platform display interface.

The “Construction Management BIM Platform” display interface will transmit GPS and inclinometer data from the top of the guided frame platform to the calculation software for processing and calculation (see Fig. 10). By comparing the actual data and relative position with the design, the platform can accurately guide on-site construction personnel in adjusting the planar position and angle of the guided frame platform to meet the design requirements.

When the suction drum's bottom is approximately 1 m from the intended position, the surveyor must check the guide frame platform's level using a spirit level to verify that the flatness aligns with the design specifications. This measure guarantees that the platform's construction and stability are high quality.

**Figure 11:**
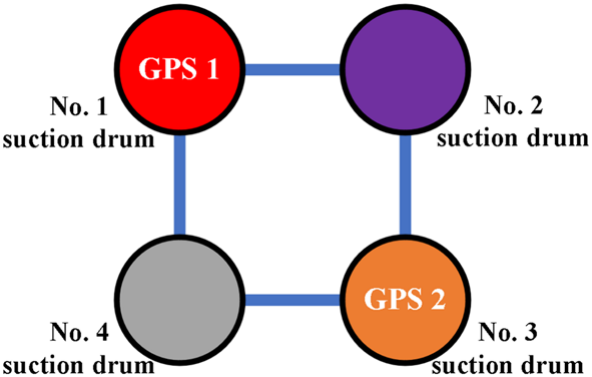
Location of monitoring equipment.

(3) Monitoring program. During the construction of the multi-barrel foundation guide frame platform, the accuracy of the monitoring information is critical in guiding the construction. The following is the monitoring program adopted:
1. Differential pressure monitoring inside and outside the drum: The pressure at the mouth of the suction pump and the pressure at the top of the suction drum are measured using pressure transducers mounted in the manifold system at the bottom of the guide frame platform. The pressure difference between the inside and outside of the drum can be calculated from the difference between the readings of these two sensors.
2. Level monitoring: There are generally two ways to measure the level of the multi-barrel base guide frame platform. One is to measure the platform inclination directly, and the other is to calculate the platform inclination by measuring the displacement height outside each barrel to achieve the purpose of smooth control. This intelligent control system adopts platform inclination monitoring to control the platform inclination. A high-precision dual-axis inclination sensor and an azimuthal electric compass are installed in the pump skid block system. A dual-axis inclination sensor can measure the X–Y-axis rotation angle to observe the guide frame platform space sinking attitude, and an azimuth electric compass can accurately determine the position and direction of the platform for the multi-barrel guide frame platform tilt on time to take leveling measures to bring convenience. At the same time, with the 3D coordinate changes of the two GPS measurement points on the top of the guide frame platform, the comparison and verification are carried out. See Fig. 11 for details.
3. Pumping flow monitoring: monitoring the dynamic change of the pumping flow of each barrel. Specific monitoring equipment can be selected according to actual needs, such as flow meters.

The aforementioned monitoring program allows for the dynamic monitoring of the pressure differential between the interior and exterior of the suction drum base (i.e., the height differential between the top and bottom of the drum cover), overall level, azimuth, and elevation of the platform, and dynamic monitoring of the pumping flow rate of each drum. This is crucial to ensure the accuracy and safety of the construction process.

**Figure 12:**
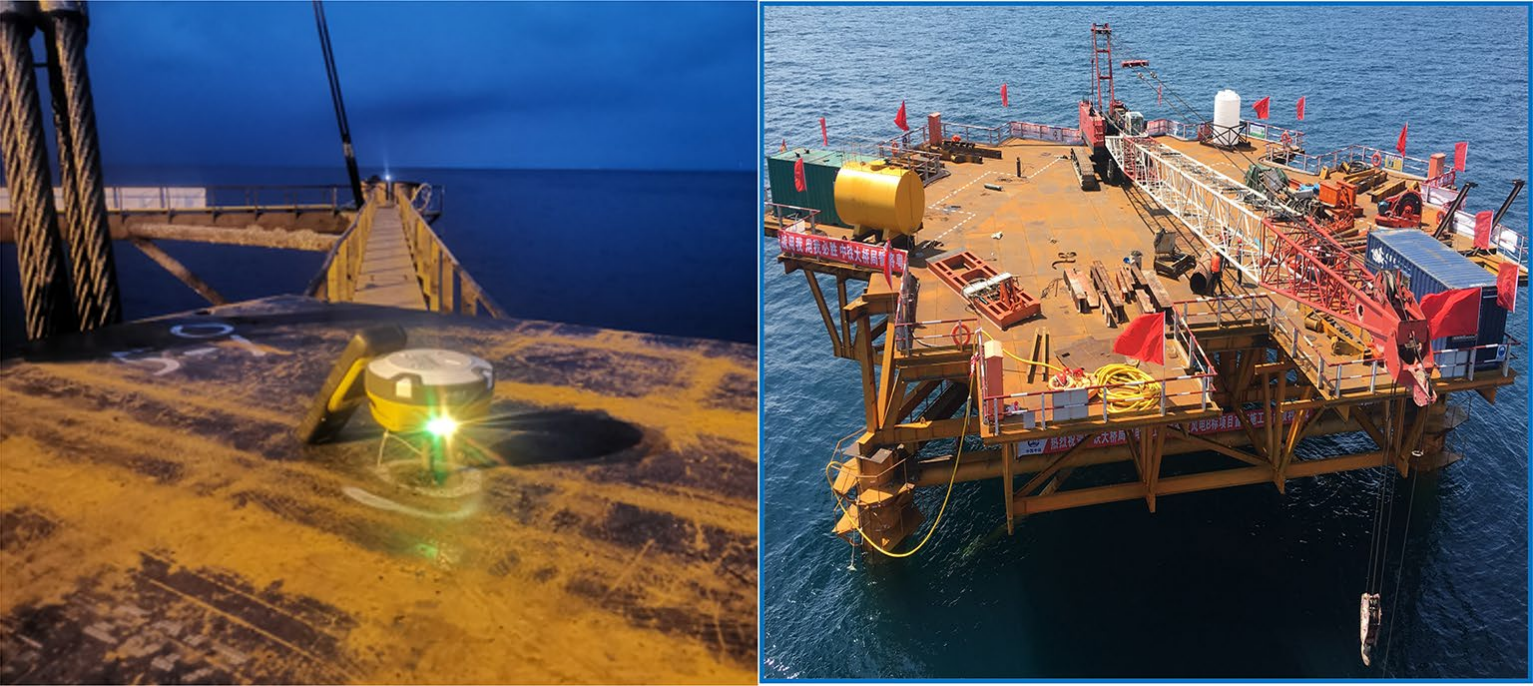
Platform top monitoring instrument and platform accurate installation diagram (photographs are derived from the project works by the author of this article).

Place the monitoring instrument on the platform's top and employ the construction management BIM platform visual measurement and positioning system to facilitate the sinking process. This will enable precise and speedy control of the position and deviation of the platform lowering and allow for ongoing dynamic observation of the platform elevation, verticality, and other critical data throughout the sinking process. These steps will support visual monitoring of the platform installation, ensuring an optimal and efficient work platform installation. Detailed information is presented in Fig. 12.

Immediately after that, the sinking depth is monitored. There are three ways to monitor the sinking depth: firstly, it is directly converted by the coordinates of GPS measurement points on the top of the guiding frame platform; secondly, it is indirectly measured by the pressure transducer; thirdly, it is directly measured by the depth transducer, which is adopted in this intelligent control system.

Finally, the sinking speed is monitored. The technicians record each time point in detail during the sinking construction, including the start and end time of exhaust sinking, the end time of the self-weight sinking, and the start and end time of pumping, etc., and record in detail the elevation of the sea level, the elevation of the foundation control point, and the depth of suction drum into the mud at each time point. The control system controls the sinking speed of the suction drum foundation by controlling the flow rate of the pump, and the speed is converted according to the sinking depth and sinking time.

### Suction drum foundation sinking process control

The control process of the suction drum foundation construction process is as follows:

1. Leveling the platform of the multi-drum guiding frame: use an inclinometer and lifting equipment to level the platform to ensure its levelness.
2. Lifting suction drum: use a crane to lift the foundation of the suction drum to the designated installation position, and gradually relax the sling to make the foundation stationary.
3. Foundation sinking into the water: slowly sink the foundation into the water until it enters the mud and begins to sink under its weight. During the period of the self-weight sinking of the foundation, it is necessary to keep the foundation stable and avoid inhaling the sediment of the external weak stratum to prevent the occurrence of the pipe surge phenomenon.
4. Standing time: after the self-weight sinks through, it should stand for some time to ensure that the suction drum forms a negative pressure that can be generated within the airtight conditions.
5. Negative pressure sinking through the construction: start the pump sled block system and start negative pressure sinking through. In the process of sinking through, use the control system to monitor the pressure difference between inside and outside the foundation in real-time. If it exceeds the limit value, the pump should be shut down in time.
6. Platform level and inclination adjustment: if the platform level exceeds the limit value, adjust the pumping volume to adjust the pressure difference between the inside and outside of each barrel, and keep the platform inclination within ± 1° to control the speed and quality of sinking through.
7. Multi-barrel guiding frame platform sinking: in the process of sinking, the relative height difference of each suction barrel shall not be more than 15 cm in order to ensure the structural safety of the platform frame.

These steps can guarantee the safety of the platform frame and suction drum foundation structure, along with the construction quality while erecting the suction drum foundation. The inclinometer and control system can monitor the platform level and pressure differential between the interior and exterior of the drums in real time, facilitating timely adjustment and control. During the construction process, it is imperative to strictly adhere to safety operation regulations to ensure the well-being of construction personnel and equipment. Furthermore, quality inspection and acceptance are necessary upon completion of construction to guarantee the durability and dependability of the suction drum foundation. This construction process can effectively manage problems and assure quality and safety.

### Analysis of real-time control of four-drum guided frame platform construction

**Figure 13:**
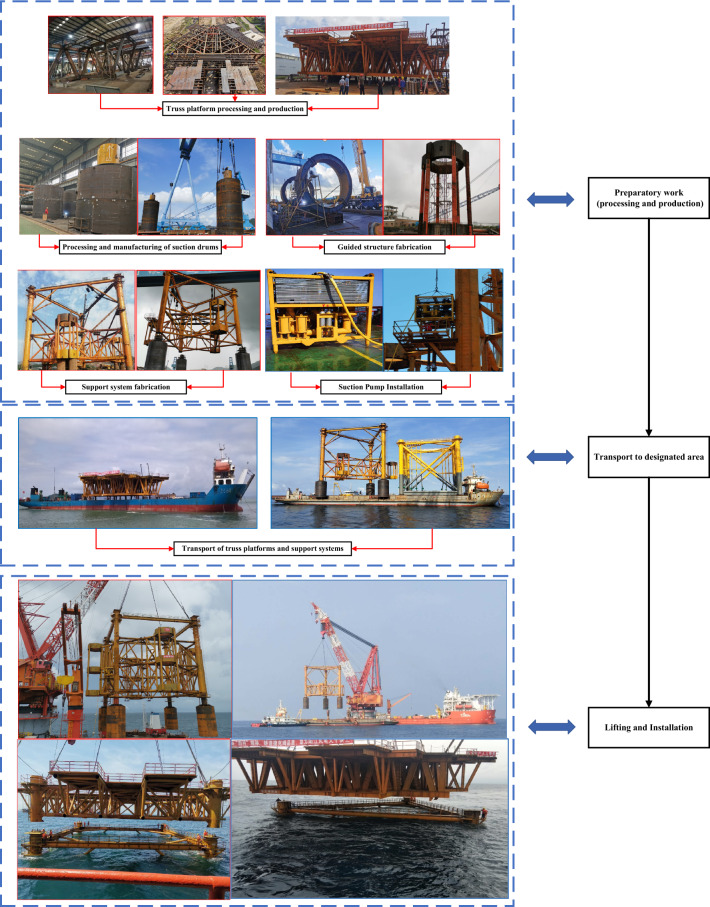
Detailed drawings of key processes (photographs are derived from the project works by the author of this article).

Using WT01, WT17, WT42, WT49, WT51, and WT52 guide frame platform suction drum foundations in the offshore wind power project in Yangjiang City, Guangdong Province, as an example, this study analyses the sinking process of the four-drum guide frame platform with the use of a control system. Figure 13 provides a detailed construction process overview.

(1)(1) Real-time analysis and control of sinking penetration with leveling: The foundation for the suction drum on the guiding frame platforms WT01, WT17, WT42, WT49, WT51, and WT52 has commenced negative-pressure sinking penetration after completing self-pressure sinking. Leveling is carried out promptly when the inclination angle during the sinking process exceeds the specification limit. The Fig. in 14a–f illustrate the inclination angle during the sinking process. The WT49 machine maintains an inclination angle of approximately 0° from the beginning until the end of the negative-pressure sinking method. In contrast, the early stage of the negative-pressure sinking of the WT01, WT17, WT42, WT51, and WT52 shows an inclination angle of *X*-direction ranging from − 0.30° to 0.26°, the inclination angle in the *Y*-direction ranges between − 0.86° and 0.30°. The inclination fluctuates significantly at the start of negative pressure penetration, owing to the foundation's instability toward the end of self-pressure penetration and the beginning of negative pressure penetration, resulting in a significant fluctuation of the initial inclination. During the descent under negative pressure, the system continuously monitors the inclination angle data and adjusts it dynamically to optimize and make timely, accurate adjustments. The platform undergoes fine-tuning before the negative pressure sinking process ends, aligning its inclination angle to 0°.

**Figure 14:**
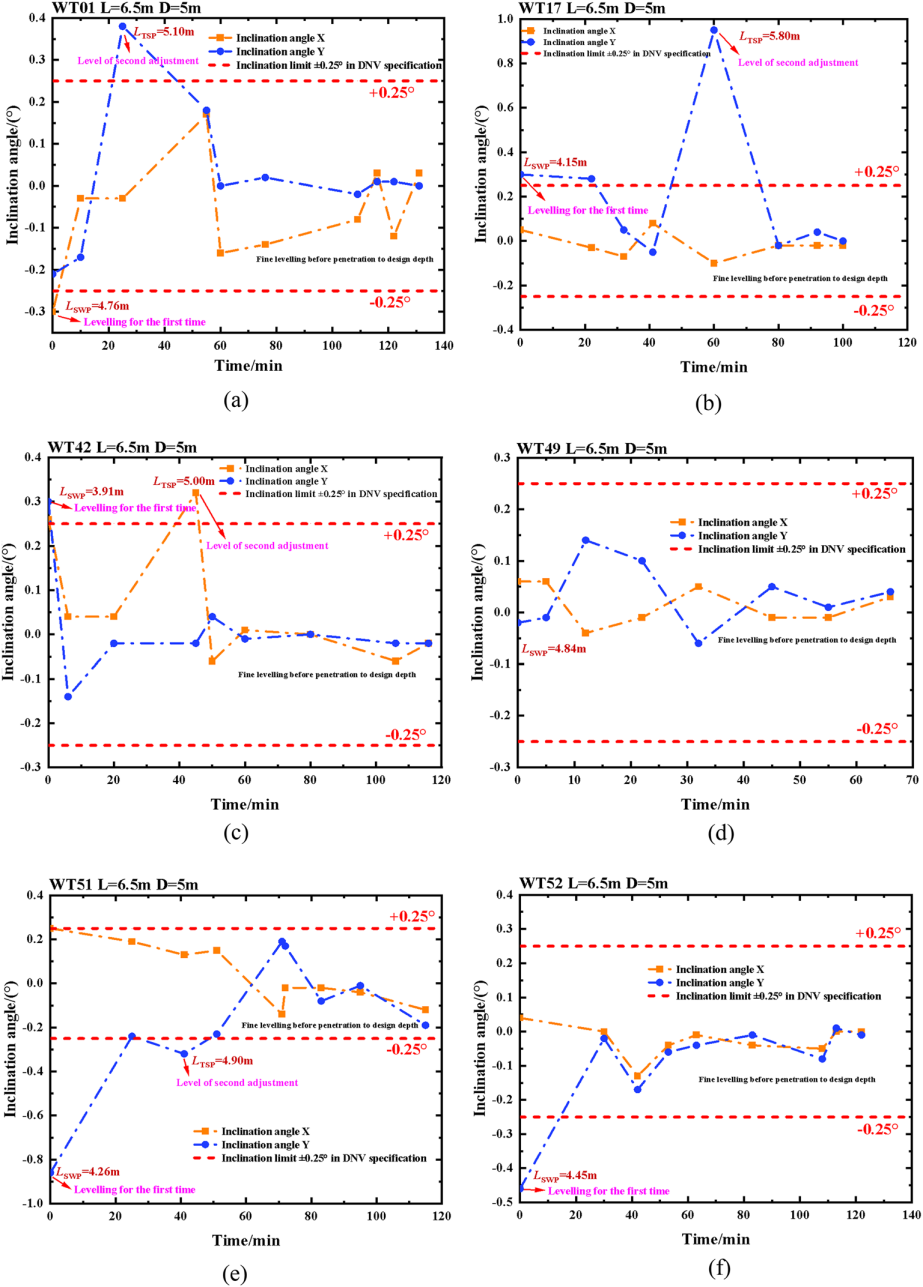
Inclination change during negative pressure sinking of four-drum guide frame platforms.

**Figure 15:**
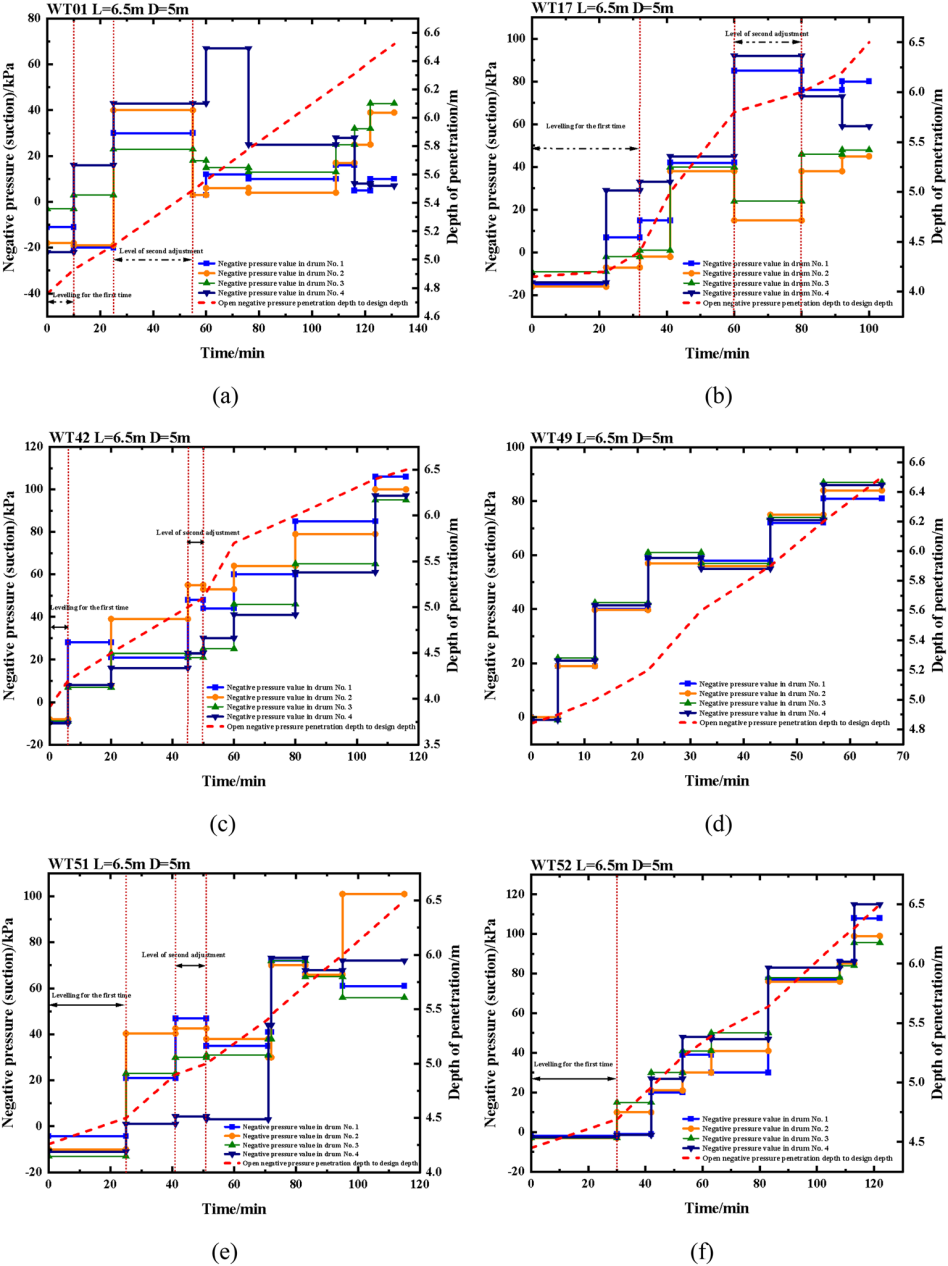
Negative pressure and depth of sinking during negative pressure sinking of four-drum guide frame platforms.

Figure 15 illustrates the changes in negative pressure and penetration depth overtime during the four-barrel guided frame platform's negative pressure penetration, as shown in (a) through (f). It is essential to demonstrate that, considering the unique properties of the suction drum foundation settling, the downward driving force generates negative pressure (suction) during the settling process, hence the necessity to designate the negative pressure value as positive. Conversely, the water injection pressure serves as the opposing force and is designated as negative. The negative pressure difference between the maximum and minimum values in each suction drum during the leveling process is more significant, implying a more vital leveling force. This is supported by the data in Fig. 14a–f.

The time taken for the initial leveling of the WT01 machine position was approximately 10 min and the maximum tilt angle in the *X* direction was -0.30°. At the beginning of the vacuum penetration, the absolute value of the *X*-direction tilt angle of -0.30° is greater than the absolute value of the $X^{\prime }$-norm value of $\pm$ 0.25°. At present, it is advisable to raise the negative pressure of No.3 and No.4 drums while lowering that of No.1 and No.2 drums for leveling purposes. After 10 min, the tilt angle of the X-direction was reduced from − 0.30° to − 0.03°, signifying the completion of leveling. The pressure in the No.1 drum increased from − 11 to − 20 kPa, thereby elevating the lifting force of the water injection by 9 kPa. Additionally, the pressure in the No.2 drum decreased from − 18 to − 19 kPa. During the leveling process, it was observed that the lifting force of water injection increased by 1 kPa when the pressure in the No.3 drum was adjusted from − 3 to 3 kPa, resulting in a negative pressure increase of 6 kPa. Similarly, the pressure in the No.4 drum was adjusted from − 22 to 16 kPa, resulting in a negative pressure increase of 38 kPa. These observations imply that the driving force provided by the negative pressure is greater than the lifting force of water injection. The second leveling process took approximately 30 min and was inclined at a maximum angle of 0.38° in the *Y* direction after 25 min of negative penetration. Notably, the absolute value of the *Y*-direction tilt angle of 0.38° is greater than the absolute value of the $Y^{\prime }$-norm value of $\pm$ 0.25°. The leveling process was completed by injecting water into drums 2 and 3. The pressure in drum 2 was adjusted from 40 to 3 kPa, increasing the lifting force of the injection by 37 kPa, while the pressure in drum 3 was adjusted from 23 to 18 kPa, increasing the lifting force of the injection by 5 kPa. Throughout the sinking process, the pressure in the drums was regularly tweaked to ensure the platform inclination angle approximated 0 degrees.

The initial leveling of the position of machine WT17 took around 32 min, with the largest angle of inclination being 0.30° in the *Y* direction. The magnitude of the *Y*-direction tilt angle at 0.30° exceeded that of the $Y^{\prime }$-norm value at the beginning of the negative pressure penetration, which was $\pm$ 0.25° in absolute terms. Leveling was done by increasing the negative pressure to drums 1 and 4 versus filling drums 2 and 3 with water. The pressure value in drum No. 1 was adjusted from − 15 to 15 kPa, and the negative pressure increased by 30 kPa; the pressure value in drum No. 4 was adjusted from − 14 to 33 kPa, and the negative pressure increased by 47 kPa. The pressure in drum No. 2 was adjusted from − 16 to − 2 kPa, and the injection lifting force increased by 14 kPa. the pressure in drum No. 3 was adjusted from − 9 to 1 kPa, and the injection lifting force increased by 9 kPa, immediately followed by an increase in suction value of 1 kPa. The second leveling process took approximately 20 min and was inclined at a maximum angle of 0.95° in the *Y* direction after 60 min of negative penetration. The absolute value of the *Y*-direction tilt angle of 0.95° is greater than the absolute value of the $Y^{\prime }$-norm value of $\pm$ 0.25°. Leveling is achieved by increasing the negative pressure to drums 1 and 4 while simultaneously reducing it to drums 2 and 3. The pressure values in Drums No. 1 and No. 4 were increased from 42 to 85 kPa and from 45 to 92 kPa, respectively. Consequently, the negative pressure rose by 43 kPa and 47 kPa accordingly. In Drum No. 2, however, the pressure value was lowered from 49 to 15 kPa, leading to a decrease in negative pressure by 34 kPa. Similarly, in Drum No. 3, the pressure value decreased from 48 to 24 kPa, resulting in a decrease in negative pressure by 24 kPa. Until the completion of the sinking process, the pressure in each drum was adjusted precisely to ensure that the platform's inclination was approximately 0°.

The initial leveling of the WT42 position lasted approximately six minutes, with an inclination of 0.26° in the *X*-direction and 0.30° in the *Y*-direction. As negative pressure began to sink in, both directions exceeded the normative value. Therefore, the leveling was performed by increasing negative pressure in drums 1 and secondarily in drums 2, 3, and 4. The pressure in drum No. 1 was changed from − 9 to 28 kPa, resulting in a 37 kPa increase in negative pressure. In drum No. 2, the pressure changed from − 8 to 8 kPa, leading to a 16 kPa increase in negative pressure. Similarly, in drum No. 3, the pressure was adjusted from − 9 to 7 kPa, causing an increase of 16 kPa in negative pressure. Lastly, the pressure in drum No. 4 was modified from − 10 to 8 kPa, which led to an increase of 18 kPa in negative pressure. The second leveling was performed after 45 min of suction sinking, taking around 5 min to complete. At this point, the maximum inclination in the *X*-direction was 0.32°. The leveling process involved injecting water into drum No. 1 and drum No. 2. The pressure in drum No. 1 was reduced from 48 to 44 kPa, which resulted in an increase of 4 kPa in the lifting force. Similarly, the pressure in drum No. 2 was lowered from 55 to 53 kPa, which resulted in a 2 kPa increase in the lifting force. During the sinking process, the pressure in the drums was adjusted to maintain the platform inclination angle at around 0°.

The position of the WT49 machine remains within specifications throughout the entire period, commencing from the start of the negative sinking penetration until the end of the sinking penetration, with only minor adjustments needed.

The initial leveling of the position of machine WT51 took around 25 min, with the largest angle of inclination being -0.86° in the *Y* direction. The magnitude of the *Y*-direction tilt angle at -0.86° exceeded that of the $Y^{\prime }$-norm value at the beginning of the negative pressure penetration, which was $\pm$ 0.25° in absolute terms. It should be taken to increase the negative pressure in drum No. 2 and drum No. 3. The pressure value in drum No. 2 is adjusted from − 10 to 40.3 kPa, and the negative pressure is increased by 50.3 kPa; the pressure value in drum No. 3 is adjusted from − 13 to 23 kPa, and the negative pressure is increased by 36 kPa. The second leveling process took approximately 10 min and was inclined at a maximum angle of − 0.32° in the *Y* direction after 41 min of negative penetration. The leveling was carried out by injecting water into drums 1 and 4. The pressure in drum 1 was adjusted from 46.9 to 35 kPa, which increased the lifting force by 11.9 kPa; the pressure in drum 4 was adjusted from 4.3 to 3 kPa, which increased the lifting force by 1.3 kPa. Until the end of the countersinking process, the pressure in the individual buckets was fine-tuned to ensure that the platform inclination angle was close to 0°.

The initial leveling of the position of machine WT52 took around 30 min, with the largest angle of inclination being -0.46° in the *Y* direction. The magnitude of the *Y*-direction tilt angle at -0.46° exceeded that of the $Y^{\prime }$-norm value at the beginning of the negative pressure penetration, which was $\pm$ 0.25° in absolute terms. It should be taken to increase the negative pressure in drum No. 2 and drum No. 3. The pressure value in drum No. 2 is adjusted from − 3 to 10 kPa, and the negative pressure is increased by 13 kPa; the pressure value in drum No. 3 is adjusted from − 3 to 15 kPa, and the negative pressure is increased by 18 kPa. Until the end of the sinking passage, the pressure values in every barrel were adjusted to guarantee that the platform inclination was close to 0°.

During the sinking process, the Six Guiding Frame Platform's suction drum base maintains a negative pressure sinking velocity that does not exceed the maximum speed limit of 2.5 cm/min. The control system timely adjusts the tilt angle within the specified range and fully satisfies the platform tilt angle requirement of not exceeding ± 1° throughout the entire sinking process, as shown in the Figs. 14 and 15. Due to the characteristics of the control system, although the platform tilt angle may not be fully zeroed, the platform lift control is highly stable with a favourable outcome. This demonstrates that the control system can effectively rectify the platform tilt angle to a secure range when the angle remains within the limit value of ± 1°.

**Figure 16:**
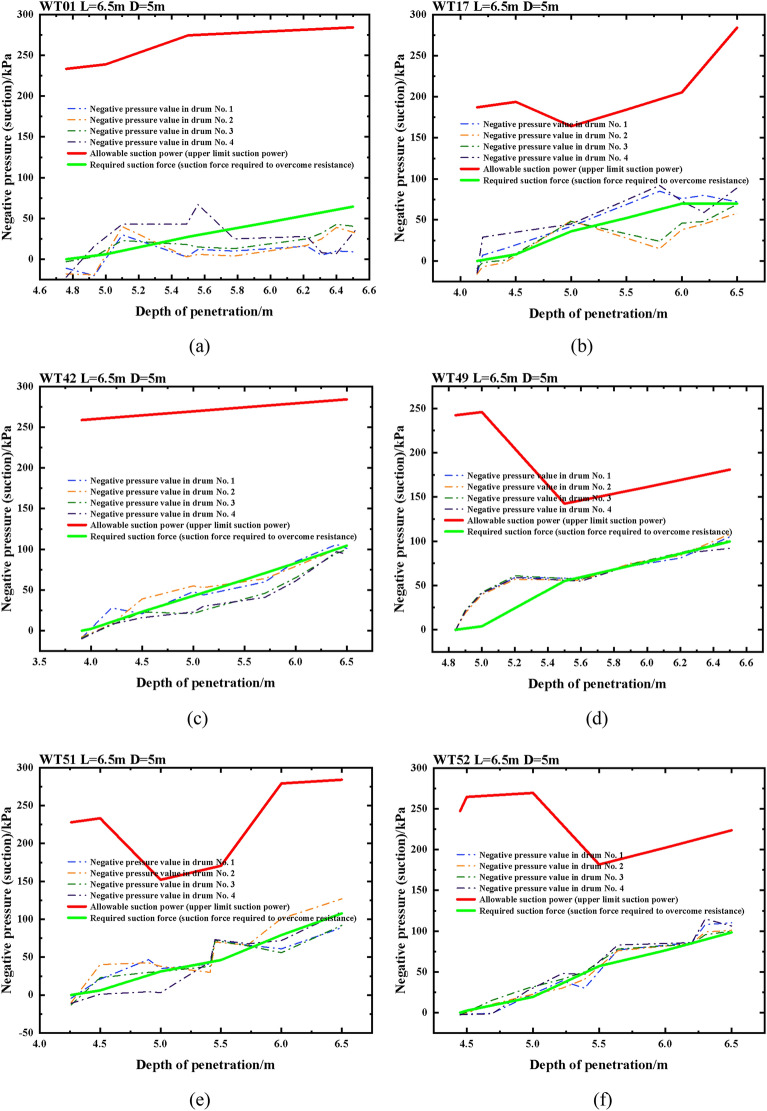
Negative pressure control curve during negative pressure sinking of a four-drum guided frame platform.

The control curves for negative pressure during the sinking process of the four-drum guided rack platform are presented in Figs. 16a–f. The graphs demonstrate that the negative pressure of each suction drum is consistently maintained within predetermined upper and lower limits throughout the sinking process. Overall, the negative pressure values fluctuate within the necessary range and are far from the acceptable negative pressure values. This redundancy provides a level of assurance in ensuring the safety of the structure.

There are two primary causes of this situation. First, it is due to soil layer inhomogeneity and variations in geological parameters. Second, the sinking process of the multi-drum guided frame platform can cause uneven sinking speeds and platform tilting, which may lead to upper load distribution adjustment.

Therefore, it is critical to emphasize strengthening monitoring and surveillance during the sinking process of the multi-drum-guided rack platform. Obtaining monitoring information promptly and adjusting the negative pressure of each suction drum based on this information is essential. This ensures the stability and safety of the suction drum foundation throughout the sinking process. At the same time, implementing appropriate monitoring and adjustment measures aids in adapting to changes in the soil layer and guaranteeing that the negative pressure of the suction drums remains within the designated range.

**Figure 17:**
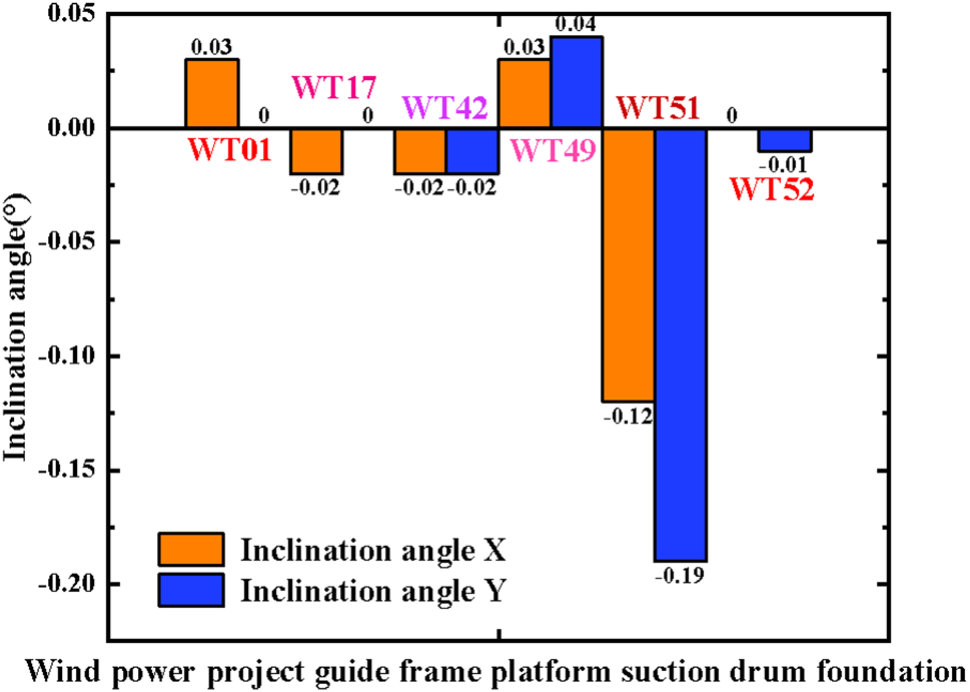
Angle of inclination for successful installation of four-drum guide frame platforms.

As demonstrated in Fig. 17, The machine platform tilt angles at the end of subsidence were all negligible. Specifically, the WT01, WT17, WT42, WT49, and WT52 machine platforms had a near-0° tilt angle, and the maximum tilt angle of the WT51 machine platform was − 0.19°, well within the prescribed limit. It is demonstrated that this set of control techniques can enable the multi-drum guide frame platform to sink smoothly, resulting in a more satisfactory sinking effect. The negative pressure of the suction drum, sinking speed, and timely adjustments based on monitoring information effectively regulate the sinking process of the multi-bucket guide frame platform. The slight variation in tilt angle demonstrates the platform's stability and the construction quality's manageability. This verifies the effectiveness of the implemented control approach in ensuring the secure submergence of the multi-drum guided frame platform.

In essence, the control system can verify that the design parameters are maintained within the designated range during the installation of the suction drum foundation. This results in improved construction efficiency and quality and decreases operational errors.

## Conclusions

This paper takes the offshore wind power project in Yangjiang City, Guangdong Province, as the background and systematically summarises the calculation of penetration resistance, penetration depth prediction, penetration feasibility analysis of the calculation method, drum strength and stability check during the construction of suction drum foundation for the guided frame platform—detailed discussion of the suction drum foundation construction process control parameters, control technology, and control standards. Moreover, take six four-pile platform suction drum foundation construction control processes as an example and compare and analyze the calculation results with the measured data. The main conclusions are as follows:

1. The penetration resistance calculation Eq. (3) originates from API specification, which is put forward by generalization and collation, applicable to both cohesive soil layer and cohesionless soil, and is the universal expression of penetration resistance of suction drum foundation, which yields the theoretical calculation results of Eq. (3) with a high degree of fit with the measured values.
2. Introducing Eq. (7) for the prediction of self-weight penetration of suction drum foundations and Eq. (8) for the prediction of suction penetration, it was concluded that the prediction of penetration depths of suction drum foundations was feasible, with error values ranging from − 0.03 to + 0.14 m.
3. The calculation formula of suction drum foundation sinking penetration resistance based on the static equilibrium method (API specification) can better reveal the changing trend of sinking penetration resistance with sinking depth. The calculation method of sinking resistance based on CPT data (Based-CPTU method) is somewhat feasible, but the recommended empirical coefficients are not fully applicable to the project engineering sea area, and the empirical coefficients *k*_*p*_ and *k*_*f*_ should be discounted for this project.
4. The strength and stability of the suction drum foundation structure are checked, and the calculated stress ratio (*UC*) and stress ratio (*UR*) are less than 1. The strength and stability of the suction drum foundation meet the design requirements.
5. In the feasibility analysis of suction drum penetration, the calculation was carried out with the example of suction drum penetration installation, and the soil layer was divided into several thickness units, and the safety coefficients K of suction drum penetration were judged to be greater than 1.25 layer by layer, and the results of the calculations showed that the method could reasonably predict the feasibility of suction drum penetration.
6. In the actual project, the leveling of the foundation of multiple drums should try to take the low water level suction drum foundation water injection to reduce the negative pressure value of the way to adjust water injection suction drums generated by the active lifting force is the effectiveness of the leveling, the greater the depth of the base of the sink through the force generated by the injection of water can be converted into the tilt of the adjustment of the effectiveness of the force. The larger the platform tilt angle, the longer the time required for leveling; the more significant the immersion depth, the larger the leveling force.
7. “Intelligent control system of suction penetration equipment integration system for accurate leveling” combined with the new technology of “construction management BIM platform” solves the core problem in the process of sinking and penetration of suction drum foundation of multi-barrel guided frame platform, i.e., leveling of suction drum foundation, and improves the leveling accuracy and the sinking and penetration accuracy of suction drum foundation. The leveling accuracy and sinking success rate are improved.
8. The Multi-drum guiding frame platform suction drum foundation sinking process should control the inclination angle within ± 1°; negative pressure sinking through the speed should be controlled within 2.5 cm/min to avoid sinking through the speed is too large for the drum of the soil body disturbance, resulting in too much soil plug. The negative pressure value should be between the permissible and required negative pressure.

## Research limitations

Our study focuses solely on the suction drum foundation for temporary foundation guided frame platforms, with a limited scope to the static load effect. It does not consider the suction drum foundation's effect under dynamic and cyclic loads. In the following study, the permanent suction bucket foundation—meaning offshore wind power foundations, wave protection structures, and similar installations—takes into account not only the impact of temporary wind and wave loads alongside seismic loads but also the deformation resistance of the structural foundation under long-term cyclic loads and the settlement of the foundation structure, as well as a range of other factors like soil liquefaction around the structural foundation. Thus, constructing fixed suction drum bases is more intricate and laborious.

## Data Availability

All data generated or analysed during the course of this study are included in this published article.
